# A protease-precursor system drives synergistic antagonism in haloarchaea

**DOI:** 10.1128/mbio.03405-25

**Published:** 2026-01-14

**Authors:** Rui Wang, Siqi Sun, Yuling Hao, Yue Ding, Xinran Jiang, Yu Jin, Demei Tu, Guoying Zheng, Jing Han, Shaoxing Chen

**Affiliations:** 1 College of Life Sciences, Anhui Normal University 644804, Wuhu, Anhui, People’s Republic of China; 2 State Key Laboratory of Microbial Diversity and Innovative Utilization, Institute of Microbiology, Chinese Academy of Sciences 85387, Beijing, People’s Republic of China; 3 State Key Laboratory of Microbial Technology, Shandong University 214177 , Qingdao, Shandong, People’s Republic of China; Albert-Ludwigs-Universitat Freiburg, Freiburg, Germany

**Keywords:** haloarchaea, antagonistic interaction, synergistic antagonism, antimicrobial peptide, halolysin, halocin, hypersaline environments

## Abstract

Antagonistic competition is a crucial survival strategy for microorganisms sharing ecological niches, playing a key role in shaping microbial communities and influencing biogeochemical cycles. Here, we report the first extracellular serine protease-dependent synergistic antagonism in archaea: a collaboration between an extracellular protease-producing strain and a precursor protein-producing strain. The serine protease secreted by the former cleaves the precursor protein released by the latter, generating an antibacterial effector molecule. This synergistic antagonism also occurs across domains (between halophilic bacteria and archaea), indicating broad ecological relevance. Using mass spectrometry and inhibition assays, we identified HFX_0892—from the model haloarchaeon *Haloferax mediterranei* ATCC 33500—as a key mediator of this process. Precursor protein HFX_0892 was cleaved by HlyR4 or other extracellular serine proteases, releasing the N-terminus of HFX_0892 (0892N), which displayed antagonistic activity against haloarchaea and bacteria. Disruption of the α-helical structure in 00892 via point mutations abolished the antagonistic activity. Furthermore, fusing the 0892N to HlyR4 did not interfere with HlyR4’s proteolytic function but conferred antibacterial activity. Gene knockout experiments revealed that HFX_0892 is not the sole antagonistic precursor protein in *H. mediterranei* ATCC 33500. This study uncovers a modular proteolytic activation mechanism that can be harnessed for antimicrobial agent development. The potential prevalence of HFX_0892-like precursors among extremophiles provides a feasible strategy for exploring structurally novel antimicrobial agents.

**IMPORTANCE:**

Antagonistic interactions are key drivers of microbial community dynamics in hypersaline environments. Here, we report, for the first time, a fan-shaped growth inhibition zone—an atypical phenotypic signature—resulting from synergistic antagonism between two halophilic archaeal species against a sensitive haloarchaeal strain. Using the model haloarchaeon *Haloferax mediterranei*, we identified a secreted precursor protein (HFX_0892) that is cleaved by a serine protease (such as HlyR4) to release an active antagonistic peptide (0892N). This novel form of archaeal interaction is defined as synergistic antagonism. The antagonistic activity of HFX_0892 is mediated by two α-helical motifs in its N-terminus, and this region can confer antimicrobial function when fused to other proteins. Notably, *H. mediterranei* encodes additional precursor proteins with potential antagonistic functions beyond HFX_0892. Our work identifies and elucidates a previously uncharacterized antagonistic interaction among archaea, providing critical insights into the complex interspecific interactions and microbial community assembly in hypersaline ecosystems.

## INTRODUCTION

Microorganisms are nearly ubiquitous on Earth, thriving even in extreme habitats such as hot springs, the deep sea, and hypersaline environments (1). Hypersaline settings—including salt lakes, saline-alkali soils, coastal salt evaporation ponds, salt mines, and high-salt foods—harbor diverse halophilic microorganisms from both the Archaea and Bacteria domains (2). Within these shared niches, microorganisms engage in complex interactions, often employing toxins to eliminate competing microbes or eukaryotic cells (3). Antagonistic competition serves as a key strategy for securing limited nutrients and space, significantly influencing community structure and biogeochemical cycling (4).

A well-known form of antagonism involves bacteriocins: ribosomally synthesized proteinaceous antibiotics secreted by bacteria to inhibit ecologically related microorganisms (5). Archaea produce analogous antimicrobials known as archaeocins, which include sulfolobicins from thermophilic archaea and halocins from halophilic lineages (6). Halocins, widely produced by rod-shaped haloarchaea, are categorized into peptide (<10 kDa) and protein (>10 kDa) forms, both typically requiring proteolytic processing for maturation, as exemplified by halocins C8 and S8 (7).

Beyond halocins, other antimicrobial mechanisms have been explored in haloarchaea. For instance, thiopeptide antibiotics—secondary metabolites with potent bioactivity—are known in many microbes (8). In *H. mediterranei*, genomic analyses identified lanthipeptide synthetase-like genes; however, their knockout did not reduce antagonistic activity (9). Subsequent transcriptomic studies confirmed high expression of lanthipeptide-related genes, although their inactivation again showed no effect on antagonism (10). These findings suggest that lanthipeptides are not the primary antagonistic agents in *H. mediterranei*. In contrast, Liang et al. identified two lanthipeptides (archalan α and β) in *Halorussus salinus* YJ-37-H, with synthetic archalan α showing potent inhibition against related haloarchaea (11).

Other studies report that carotenoids from haloarchaeal membranes exhibit moderate antibacterial activity against common pathogens such as *Escherichia coli* and *Staphylococcus aureus* (12). More recently, Strock et al. identified peptidoglycan hydrolases (PGHs) in about 5% of haloarchaeal genomes; two such enzymes from *Halogramum salarium* inhibited the halophilic bacterium *Halalkalibacterium halodurans* (13). However, neither lanthipeptides, carotenoids, nor PGHs appear to constitute the principal means by which haloarchaea antagonize ecologically similar competitors in high-salt environments.

Thus, while previous research has largely focused on characterizing known antagonistic molecules (7, 14–16), it remains unclear whether haloarchaea deploy synergistic interactions to generate novel antimicrobial activities. Proteases are known to contribute to the maturation of antimicrobial proteins like halocins (17), and many haloarchaea secrete extracellular proteases (18). This raises an intriguing possibility: could such proteases activate latent precursor proteins into functional antagonistic effectors through proteolytic cleavage?

In this study, we identified a striking synergistic antagonism between extracellular protease-producing and non-producing haloarchaeal strains. Using a combination of *in vivo* (gene knockout and complementation) and *in vitro* (heterologous expression, protein purification, refolding, and activity assays) approaches, we demonstrated that an extracellular serine protease cleaves a latent precursor protein, HFX_0892, to generate an active antagonistic effector. Our subsequent investigations (i) mapped the spatial dynamics of synergistic inhibition by quantifying effective antagonistic distance, (ii) established a pipeline for isolating and characterizing latent antagonistic precursors, (iii) deciphered the proteolytic processing of HFX_0892, and (iv) screened the antimicrobial spectrum of the activated effector.

This work provides the first evidence of extracellular protease-mediated synergistic antagonism in prokaryotes and establishes a comprehensive mechanistic framework for this phenomenon. Our findings redefine understanding of microbial competition in extreme environments, underscoring the role of proteolytic activation in precursor-effector systems among archaea.

## RESULTS

### Discovery of the synergistic antagonism

Antagonistic interactions are widespread among microorganisms in natural environments. Our previous study demonstrated that halolysin HlyR4, an extracellular serine protease from *H. mediterranei* ATCC 33500, is involved in the antagonistic activity against various haloarchaeal strains (19). However, HlyR4 itself exhibits no direct inhibitory effect, suggesting that it may function by cleaving a precursor protein—such as HalH4—to generate an active antimicrobial peptide (20).

**Fig 1:**
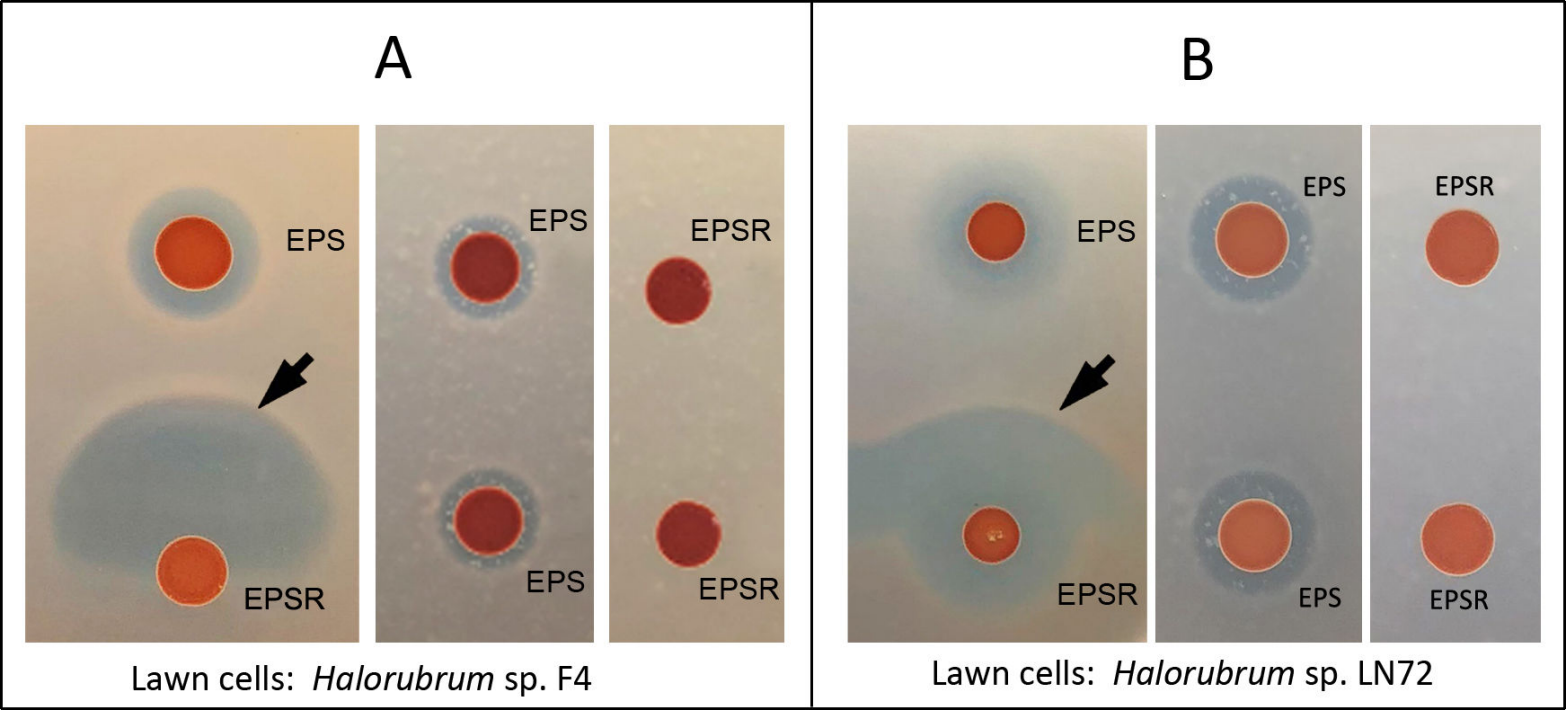
Discovery of the synergistic antagonism between two haloarchaeal strains on *Halorubrum* agar plates. The synergistic antagonism phenomenon was detected between strains EPS and EPSR (black arrows), specifically on the side adjacent to the colony of strain EPSR. The fan-shaped inhibition zone was observed on the indicator plate of *Halorubrum* sp. strains F4 (**A**) and LN72 (**B**). Strain EPS exhibits inhibition activity against strains F4 and LN72, whereas strain EPSR only presents the inhibition activity against strain LN72. Cells were cultivated on AS-168 medium supplemented with uracil (50 μg·mL^−1^) for 30 days at 42°C. EPS, *Hfx. mediterranei* strain EPS (EPS for short); EPSR, *Hfx. mediterranei* strain EPSR (EPSR for short), and the halolysin-deficient strain.

**Fig 2:**
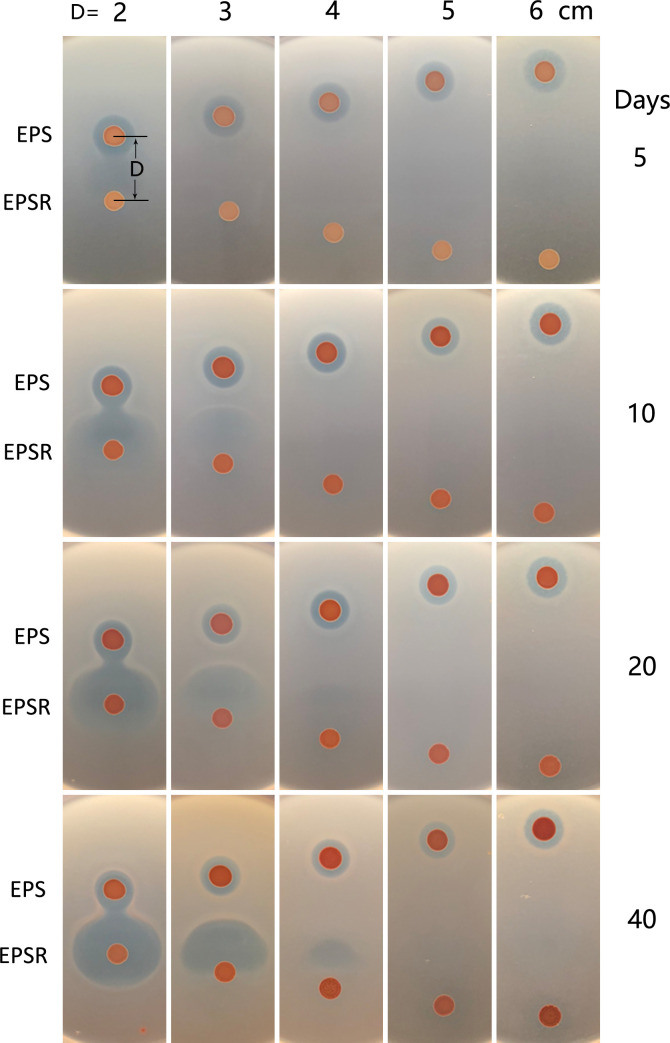
The influence of distance in the formation of synergistic antagonism. Letter “D” represents the distance between the centers of EPS and EPSR colonies. Strains EPS and EPSR were cultivated under the same conditions as described in Fig. 1. Days, cultivation days. Lawn cells: *Halorubrum* sp. F4.

To test this hypothesis, we co-cultivated strain EPS and its HlyR4-deficient derivative, EPSR, on agar plate lawns with *Halorubrum* sp. LN72 or *Halorubrum* sp. F4 as indicator strains. A distinct fan-shaped inhibition zone (FSIZ) formed adjacent to the EPSR colony on both lawn types (Fig. 1). The timing of FSIZ appearance depended on the distance between the EPS and EPSR colonies: closer proximity led to earlier formation. Notably, on LN72 lawns, a recognizable FSIZ was observed after 6 days (Fig. S1), whereas no such zone developed when colonies were spaced more than 5 cm apart, even after extended incubation (Fig. 2). FSIZ emergence occurred earlier on LN72 than on F4 plates. Additionally, a circular inhibition zone formed exclusively around the EPS colony on LN72 lawns (Fig. S1), indicating higher sensitivity of strain LN72 compared to F4. Phylogenetic analysis further distinguished these strains, revealing that LN72 clusters closely with *Halorubrum aquaticum*, whereas F4 occupies a distinct clade with uncertain affiliation (Fig. S2).

The formation of FSIZ required physical proximity between EPS and EPSR colonies. When the two strains were cultured without contact, no typical FSIZ developed, ruling out the involvement of volatile compounds in this interaction (Fig. S3).

### Identification of the antagonistic effector molecule

**Fig 3:**
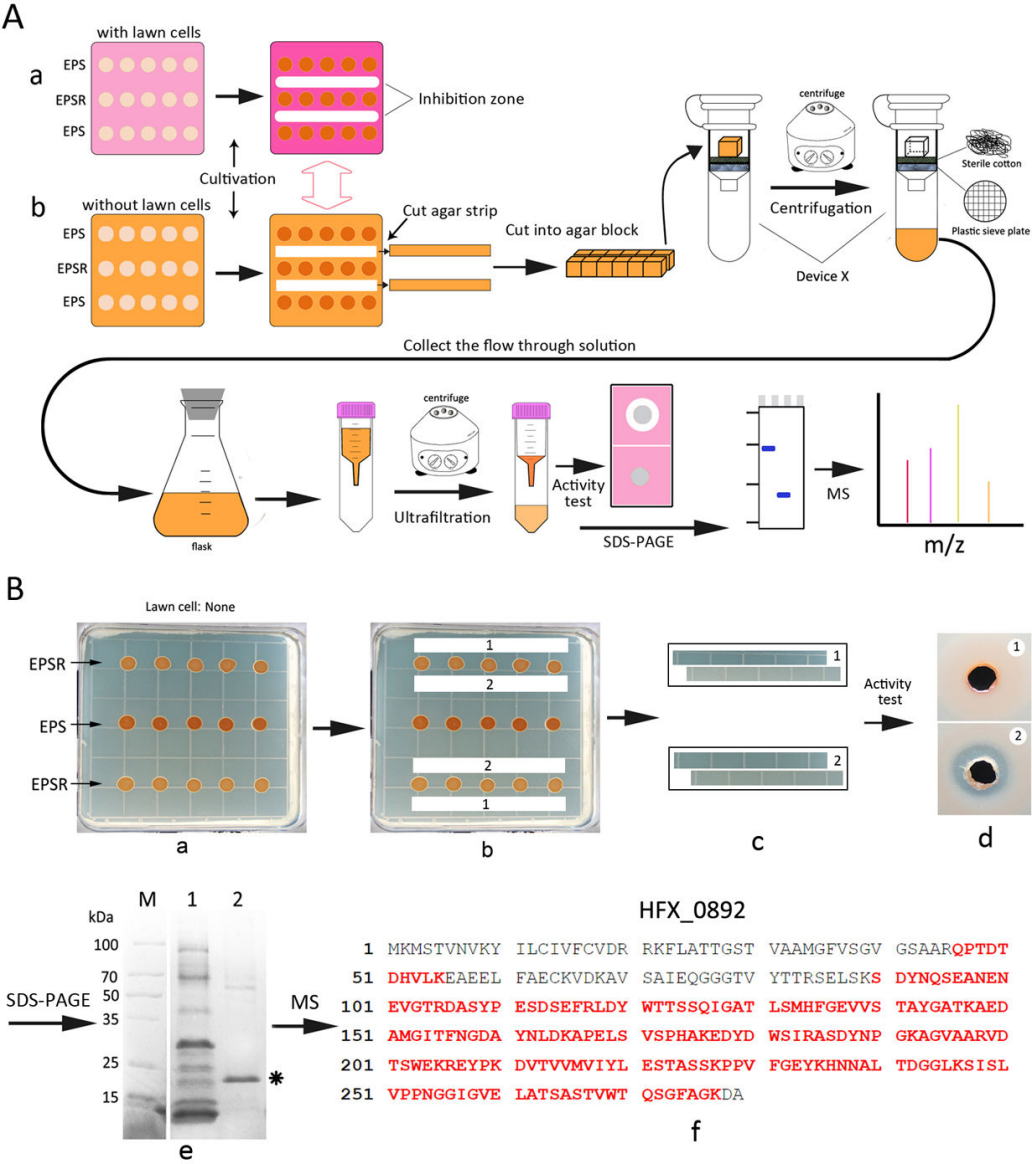
Isolation and identification of antimicrobial peptide(s) in the fan-shaped inhibition zone between strains EPS and EPSR. (**A**) The general schematic diagram for isolation and identification of antimicrobial peptide(s) with synergistic antagonism activity. The indicating plate contained lawn cells (a), whereas the experimental plate (b) did not. The cell suspension of strains *Hfx. mediterranei* EPS and EPSR was dropped onto the indicating and experimental plates in equal volume. When the fan-shaped inhibition zones appeared on the indicating plates, the agar strips at the corresponding places on the experimental plates were cut out with a sterilized blade for further antimicrobial peptide(s) extraction. The device X was designed and made in this study by replacing the filler in the column with sterile cotton. (**B**) Experimental process for determining the antimicrobial peptide(s) with synergistic antagonism activity secreted by *Hfx. mediterranei*. Based on the appearance of the fan-shaped inhibition zones between strains EPS and EPSR on the indicating plate (data not shown), agar strips at the corresponding region on the experimental plate without lawn cells (b) were cut and picked out for liquid extraction (c). The solution extracted from the agar strips was subjected to antihaloarchaeal activity detection on LN72 plate (d). Meanwhile, the solution was also subjected to sodium dodecyl sulfate polyacrylamide gel electrophoresis (SDS-PAGE) after precipitation with 40% trichloroacetic acid (TCA). The protein molecular standard is shown on the left (e). The control was numbered in digit 1 (“1”), whereas the experimental sample was numbered in digit 2 (“2”) (e). The protein band (*) (e) was subjected to mass spectrometry (MS) following in-gel trypsin digestion, and the MS results are shown in (f). Through MS analysis, the matched peptides are shown in bold red letters (f).

Our work suggested that EMs exerting antagonistic activity were present within the FSIZ; thus, we developed an isolation strategy specifically for the separation of EMs from agar (Fig. 3A). When multiple FSIZs formed in close proximity, they merged into a continuous inhibition belt on lawns of sensitive cells (Fig. 3Ba). EM-containing mixtures were collected from analogous non-lawn agar regions corresponding to these inhibition belts (Fig. 3Bb and 3c). The antagonistic activity of the extracted mixture was confirmed by spotting it onto *Halorubrum* sp. LN72 lawns (Fig. 3Bd), followed by trichloroacetic acid (TCA) precipitation and SDS-PAGE analysis (Fig. 3Bd).

By integrating the annotated genome of *H. mediterranei* ATCC 33500 with mass spectrometry (MS) data, we identified a peptide exhibiting 85% sequence match to a segment of the protein HFX_0892 (Fig. 3Be). Although these findings implicated HFX_0892 in antagonistic activity, its native biological function remained uncharacterized. We therefore designated this protein HmHapP (*H. mediterranei* haloarchaeal antagonistic precursor protein).

### Synergistic antagonism through proteolytic activation of precursor HFX_0892 by halolysin HlyR4

**Fig 4:**
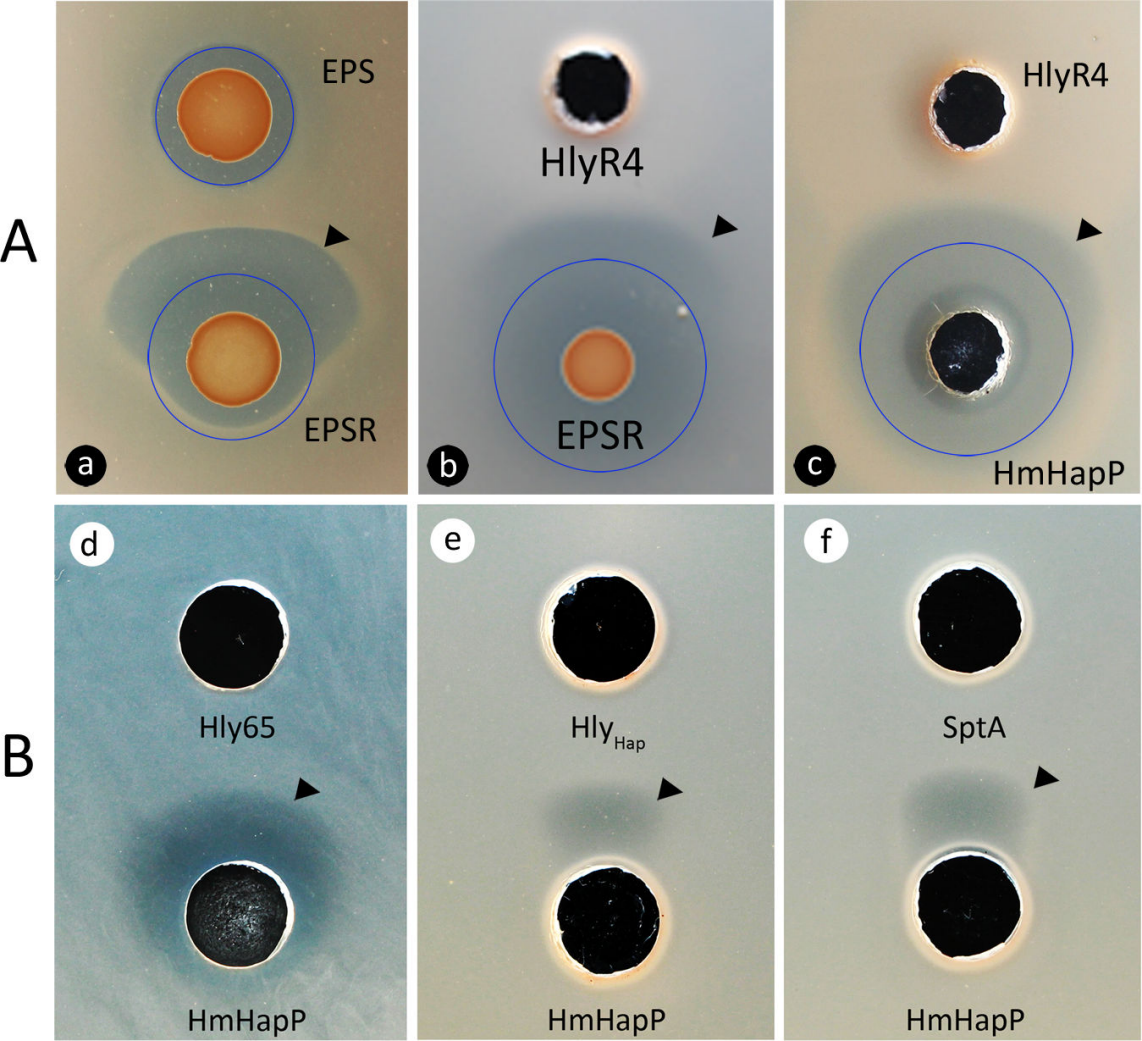
Involvement of halolysins and HFX_0892 (namely HmHapP) in the formation of synergistic antagonism. (**A**) The synergistic antagonism interaction between proteins HmHapP and halolysin HlyR4 was similar to the effect between strains EPS and EPSR on the LN72 agar plate. (a) The prototype of synergistic antagonism. (b) Strain EPS was replaced by halolysin HlyR4. (c) Halolysin HlyR4 and HmHapP were used to replace strains EPS and EPSR, respectively. (**B**) Halolysins Hly65 (d), Hly_Hap_ (e), and SptA (f) like HlyR4 can also interact with HmHapP in generating a fan-shaped inhibition zone. All these inhibition activity detections were carried out on LN72 agar plates. Proteins were expressed in *E. coli* BL21 (DE3) and purified through Ni-NTA affinity chromatography. Protein refolding and ultrafiltration were performed prior to determination of their activity. The protein concentration was nearly 1.0 mg·mL^−1^. The filled black triangles show the synergistic antagonism interaction region. The blue circles are drawn in aid of judging the synergistic antagonism.

No synergistic antagonism was observed between two EPSR strains (Fig. 1), confirming that *hlyR4* is required for the generation of effector molecules (EMs). To determine whether its gene product, the extracellular protease, HlyR4, could functionally substitute for strain EPS, we heterologously expressed, purified, and refolded HlyR4 in *Escherichia coli* (Fig. S4). The recombinant protein successfully restored synergistic antagonism when combined with EPSR on LN72 lawns (Fig. 4Ab).

HlyR4—the first genetically characterized halolysin (21)—exhibited optimal enzyme activity at 45 °C, 4.0 M NaCl, and pH 9.0 (Fig. S5a through c). Its activity was strongly inhibited by EDTA, DTT, Zn^2+^, and Cu^2+^, and moderately suppressed by Fe^2+^, K^+^, Ca^2+^, and Mg^2+^ (Fig. S5d and e). Complete inhibition by PMSF (10 mM) confirmed its classification as a serine protease. Kinetic analysis yielded the following parameters: *V*_max_ = 2,093.58 ± 86.32 U·mg^−1^, *K*_m_ = 1.91 ± 0.19 mM, and *K*_cat_ = 1,125.76 ± 112.80 s^−1^ (Fig. S5f).

We next evaluated the role of the precursor protein HmHapP (HFX_0892). Heterologously expressed and refolded HmHapP (Fig. S6) functionally replaced strain EPSR in synergistic inhibition assays (Fig. 4A and C). Notably, other reported halolysins—including Hly65 from *Halorubellus* sp. PRR65 (Fig. S7), HlyHap from *Haladaptatus* sp. DYF46 (Fig. S8), and SptA from *Natrinema gari* J7-2 (Fig. S9)—also elicited synergistic antagonism when paired with HmHapP (Fig. 4Bd through Bf).

To assess the antimicrobial spectrum of the HmHapP–HlyR4 system, the protein mixture was tested against a panel of haloarchaeal and bacterial strains (Table 3). The combination exhibited inhibitory activity against several haloarchaeal genera—*Haloarcula*, *Halorubrum*, *Halolamina*, *Haloparvum*, and *Halobellus*—but showed no activity against bacteria.

### Broad conservation of synergistic antagonism across halophilic strains

**Fig 5:**
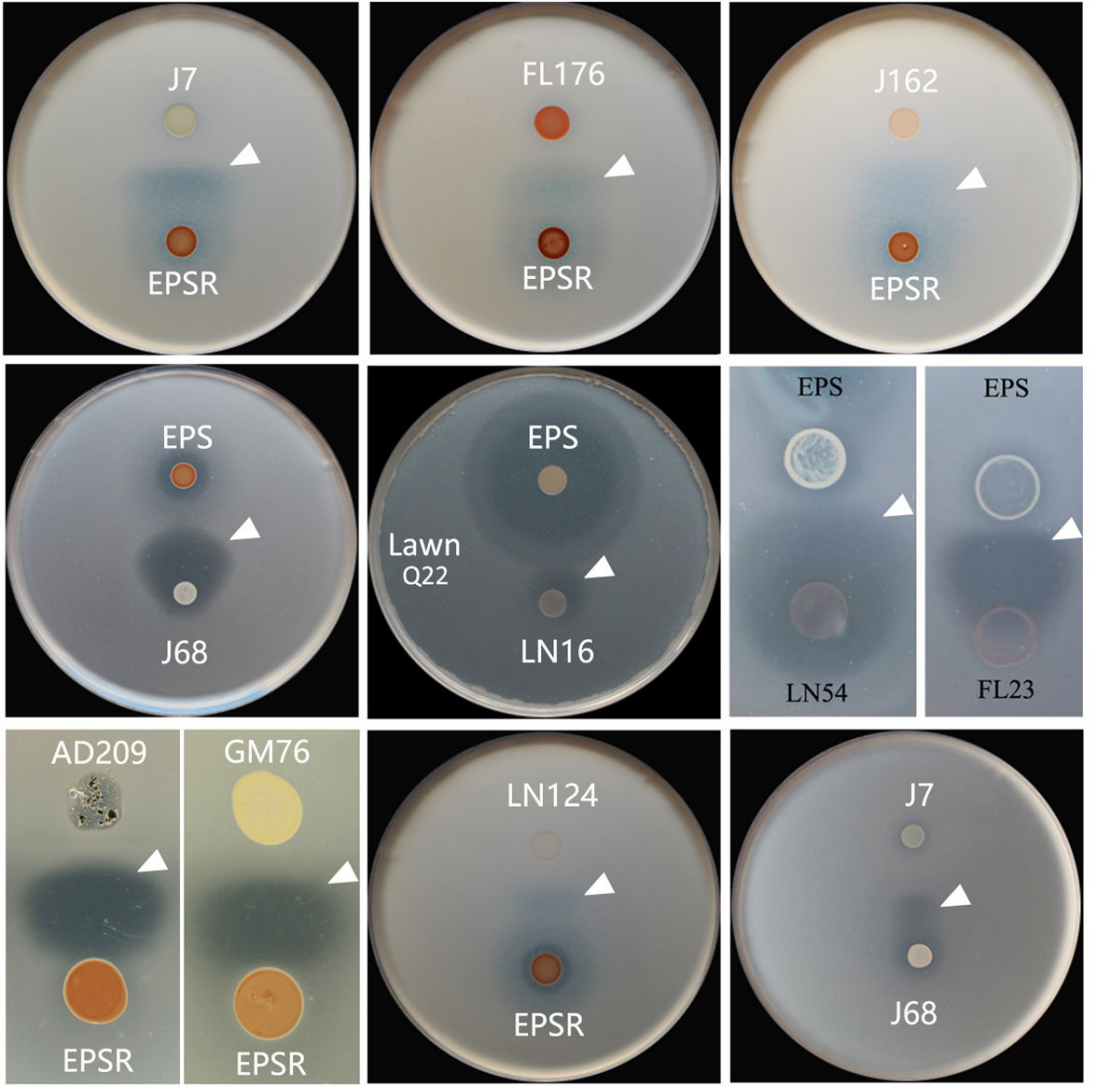
Screening of synergistic antagonism interaction between different haloarchaeal strains. Haloarchaeal strains: J7, *Natrialba* sp. J7; FL176, *Haloarchaeobius* sp. FL176; J162, *Halococcus* sp. J162; J68, *Haloterrigena* sp. J68; LN16, *Haloterrigena* sp. LN16; LN54, *Natronoarchaeum* sp. LN54; FL23, *Halorubrum* sp. FL23. EPSR, *Haloferax mediterranei* EPSR; EPS, *Haloferax mediterranei* EPS; bacterial strains: LN124, *Pseudomonas* sp. LN124; AD209, *Saccharomonospora* sp. AD209; GM76, *Halobacillus* sp. GM76. Lawn cells: *Halorubrum* sp. LN72 or *Haloferax* sp. Q22. The white filled triangles show the fan-shaped inhibition zone.

Having established that halolysins Hly65, HlyHap, and SptA can functionally substitute for HlyR4 in synergistic antagonism, we next asked whether other halophilic microorganisms could replace strain EPS or EPSR in this interaction. Strain substitution assays revealed that several haloarchaeal strains—*Natrialba* sp. J7, *Haloarchaeobius* sp. FL176, *Halococcus* sp. J162, and *Halococcus* sp. J510 (Fig. S10)—as well as bacterial strains *Pseudomonas* sp. LN124, *Saccharomonospora* sp. AD209, and *Halobacillus* sp. GM76 could effectively substitute for strain EPS (Fig. 5; Table 4). All of these EPS-substituting strains were confirmed to produce extracellular proteases (Fig. S10).

In contrast, only haloarchaeal strains—*Haloterrigena* sp. J68, *Haloterrigena* sp. LN16, *Natronoarchaeum* sp. LN54, and *Halorubrum* sp. FL23—were able to replace strain EPSR (Fig. 5; Table 4). No bacterial substitutes for EPSR were identified in this study, although it remains plausible that such strains exist in nature.

Notably, several of the substituting strains were co-isolated from the same environmental samples. For example, EPS-substituting strains J7, J162, and J510, along with EPSR-substituting strain J68, all originated from a Bolivian salt sample. A similar pattern was observed among strains from Yunnan salt mines (LN124 vs. LN16, and LN54) and from salted seaweed (FL176 vs. FL23). The repeated co-occurrence of functional partner strains in the same habitats suggests that synergistic antagonism may be a common and ecologically relevant interaction within natural microbial communities.

### HmHapP was cleaved by HlyR4

**Fig 6:**
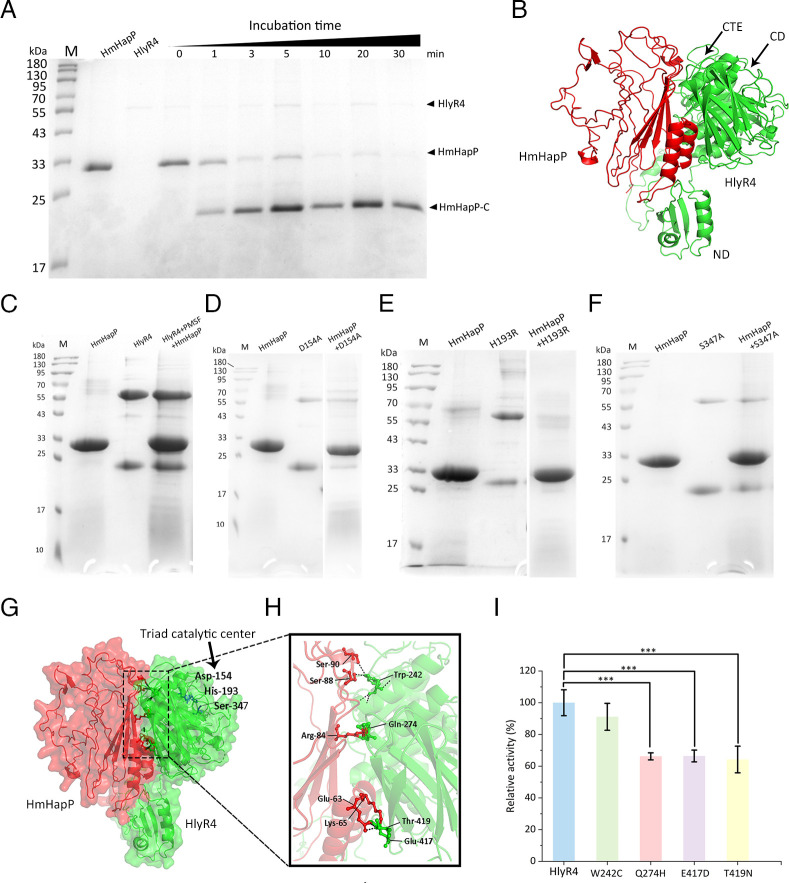
Cleavage of HmHapP by HlyR4 and its catalytic triad point mutation mutants. (**A**) Time course of HmHapP cleavage by HlyR4. The band of the HmHapP-C was determined through mass spectrometry (MS). The protein molecular standard is shown on the left. (**B**) The predicted simulation structure of the interaction between HmHapP (red) and HlyR4 (green) using AlphaFold3 (https://alphafold.com/) with cartoon mode. ND, N-terminal domain; CD, Core domain or catalytic domain, where the catalytic triad is located; CTE, C-terminal extension. (**C**) Impact of the phenylmethanesulfonyl fluoride (PMSF) on the cleavage activity. Three catalytic triad point mutation mutants, D154A (**D**), H193R (**E**), and S347A (**F**) were applied to cleave the HmHapP. (**G**) Structure prediction (cartoon and surface mode) to show the catalytic triad of halolysin HlyR4, Asp^154^-His^193^-Ser^347^ (black arrow). (**H**) Partial enlarged detail to show the possible amino acid residues in the contact surface between HmHapP and HlyR4. (**I**) Influence of the amino acid residues of HlyR4 in the contact surface on cleavage activity. W242C, Q274H, E417D, and T419N are four point-mutation mutants of HlyR4. *** *P*<0.001.

Neither HmHapP nor HlyR4 exhibited intrinsic antimicrobial activity when tested individually; however, their combination showed strong antagonistic effects against sensitive haloarchaea (Table 3). To elucidate the proteolytic mechanism underlying this synergy, we incubated HmHapP with decreasing concentrations of HlyR4 and observed time-dependent cleavage of HmHapP, accompanied by the appearance of a new peptide band (Fig. 6A). Mass spectrometry confirmed that this product, designated HmHapP-C, was identical to the bioactive peptide identified earlier (Fig. 3). Additional halolysins—HlyHap, SptA, and Hly65—also cleaved HmHapP to generate HmHapP-C (Fig. S11), suggesting a conserved cleavage site and mode of action among these proteases.

Structural modeling using AlphaFold3 indicated that HlyR4 forms a hydrophobic pocket that accommodates HmHapP (Fig. 6B). To probe the catalytic mechanism, we mutated Asp^154^ in the catalytic triad to alanine (D154A), which completely abolished proteolytic activity (Fig. 6D). Similar loss-of-function effects were observed with mutations at the other two residues of the triad (Fig. 6E and F), confirming that all three are essential for cleavage.

We further investigated potential substrate-binding residues on HlyR4 using AlphaFold-Multimer. Four interface residues—Trp^242^, Gln^274^, Glu^417^, and Thr^419^—were predicted to interact with HmHapP (Fig. S12). Mutagenesis to W242C, Q274H, E417D, and T419N (Fig. S13) resulted in only partial reductions in cleavage efficiency (10%–30% decrease compared to wild-type, Fig. 6I), indicating that these residues contribute to, but are not critical for, proteolytic function.

### The N-terminus of HmHapP is essential for its antihaloarchaeal activity

**Fig 7:**
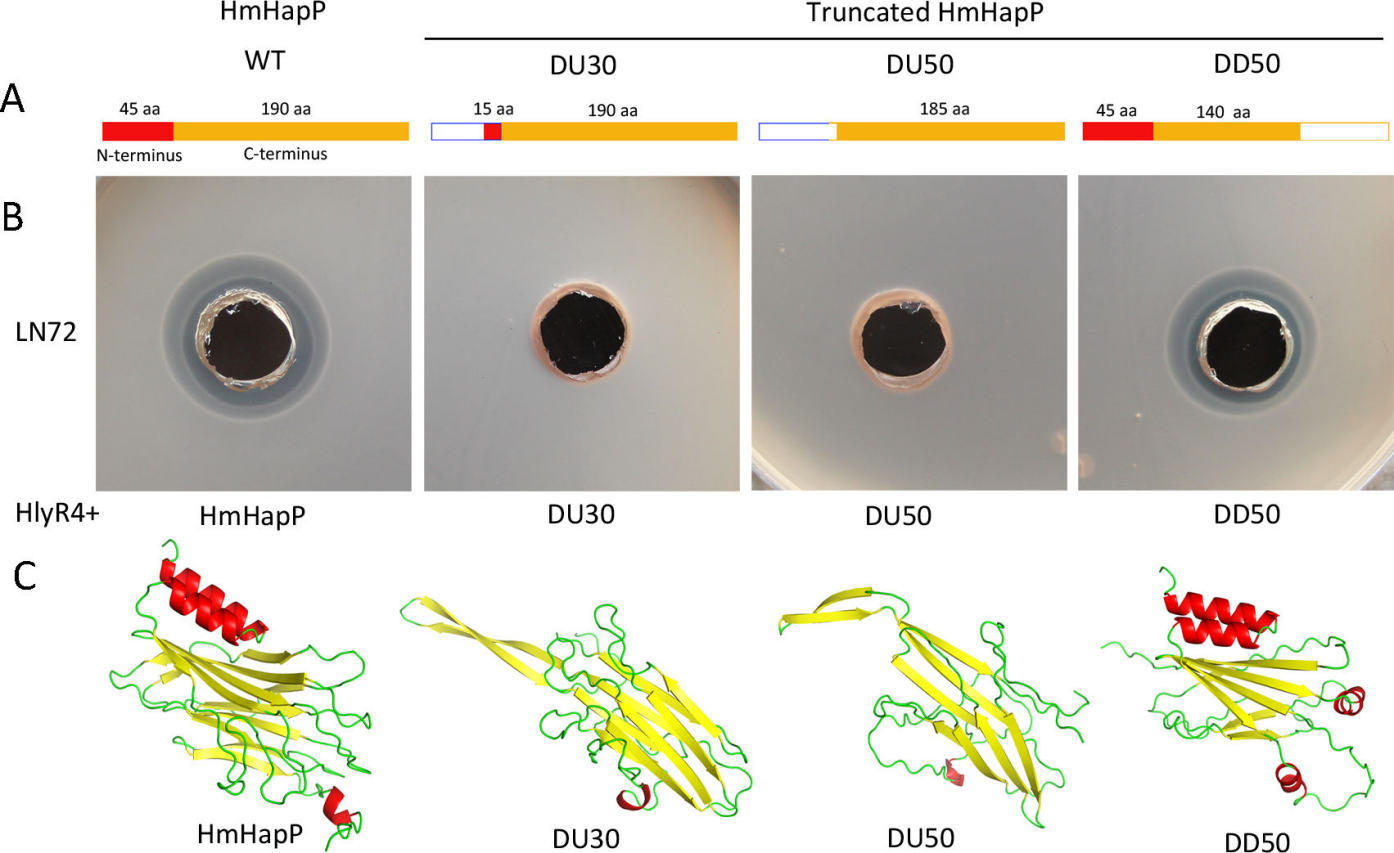
Antihaloarchaeal activity of the N- or C-terminus of HmHapP against cells of haloarchaeal strain LN72. (**A**) Schematic diagram of the design of truncated mutants. HmHapP, the wild type; DU30, deletion of the 30 aa from the N-terminus; DU50, deletion of the 50 aa from the N-terminus; DD50, deletion of the 50 aa from the C-terminus. Red-filled rectangles show the N-terminus, and the width of the rectangle corresponds to the number of amino acids. The empty boxes show the deletion region. (**B**) HlyR4, HmHapP (WT), DU30, DU50, and DD50 were expressed in *E. coli* BL21 (DE3) and purified using Ni-NTA affinity chromatography. Before performing the inhibition activity detection, refolding and concentration were required. The refolded proteins, HmHapP, DU30, DU50, and DD50, were mixed with HlyR4, respectively. Then, the mixture was dropped into the holes punched on the agar plate with the lawn cells of strain LN72. (**C**) Structure prediction using AlphaFold2 (https://alphafold.com/) to show the structure changes among the HmHapP and its truncated mutants after truncation.

To determine whether the antimicrobial activity of HmHapP cleavage resides in the C-terminal peptide HmHapP-C or another fragment, we analyzed a series of truncation mutants. HlyR4 was used to cleave two N-terminal truncation variants (DU30 and DU50) and one C-terminal truncation mutant (DD50; Fig. S14). While DU30 and DU50 lost all inhibitory activity after proteolysis, DD50 retained clear antagonistic function against *Halorubrum* sp. LN72 (Fig. 7A and B). These results indicate that the N-terminal region of HmHapP is essential for antimicrobial activity. Structural prediction via AlphaFold3 further revealed that this functional N-terminal domain (HmHapP-N) contains two characteristic α-helices (Fig. 7C).

**Fig 8:**
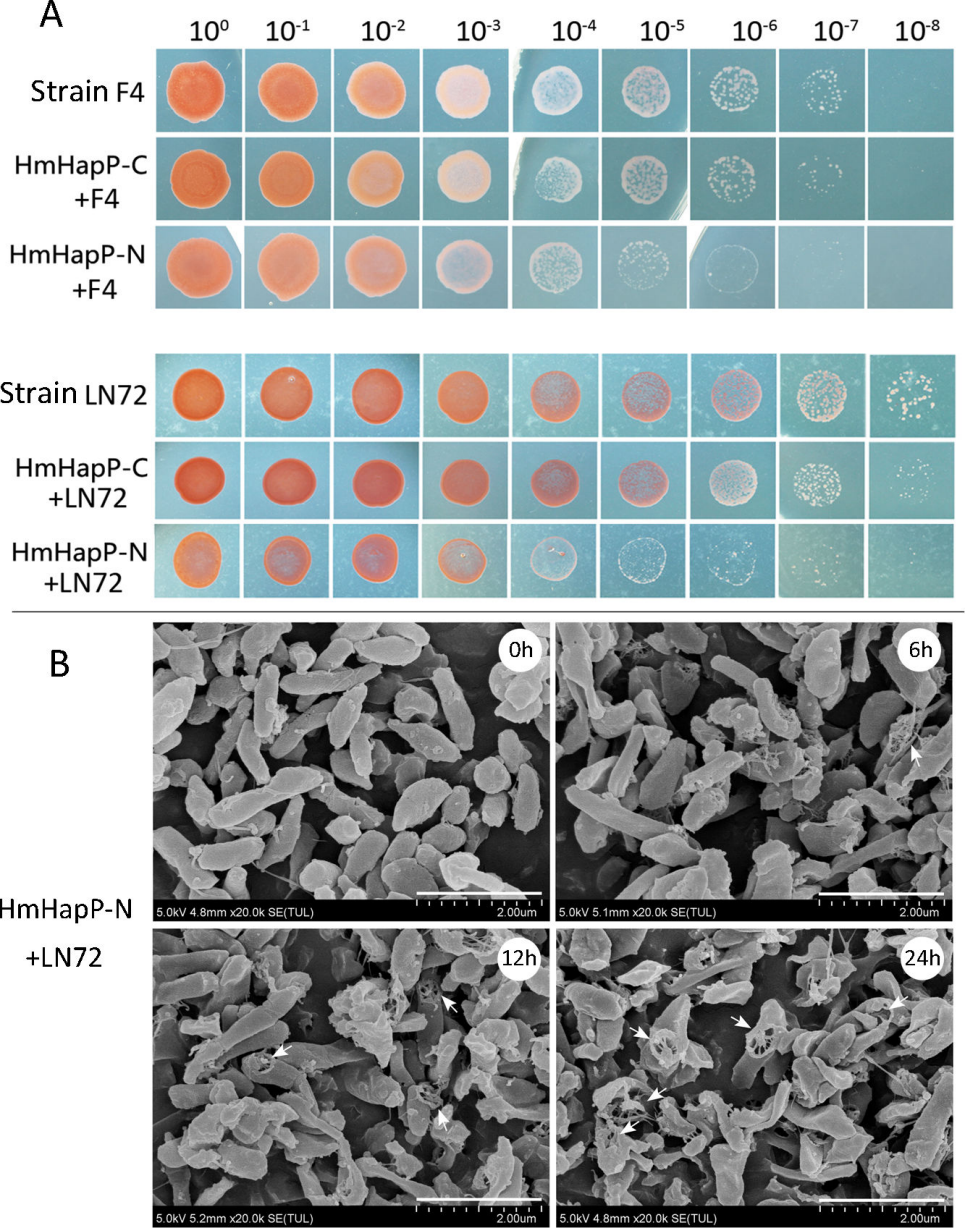
Inhibition activity of the HmHapP-N against haloarchaeal cells. (**A**) Comparison of the inhibition activity of the HmHapP-C and HmHapP-N against lawn cells of strains F4 and LN72. Cells on logarithmic phase (OD_600_≈1.0) were used, and cells treated by HmHapP-C or HmHapP-N were cultivated for 10 days at 42 °C. The dilution gradient was shown on the top. (**B**) Scanning electron microscopy (SEM) was adopted to assess the effect of the peptide HmHapP-N, the N-terminal 45 aa of HmHapP, on the cell morphology of strain LN72 over time. The white arrows show the ruptured cells. Bar, 2 μm.

To directly assess the bioactivity of HmHapP-N, we heterologously expressed and purified both HmHapP-N (Fig. S15) and HmHapP-C (Fig. S16) in *E. coli*. In gradient dilution assays, only HmHapP-N exhibited dose-dependent bacteriolytic activity, whereas HmHapP-C showed no inhibitory effect (Fig. 8A).

Scanning electron microscopy (SEM) confirmed the haloarchaeacidal effect of HmHapP-N, showing a time-dependent increase in lysed cells (Fig. 8B). High-magnification images revealed that cell disruption was associated with a distinctive wheel-like structural defect on the cell surface, rather than the classical bomb-crater morphology (Fig. S17).

### The α-helical domains mediate the antimicrobial activity of HmHapP-N

**Fig 9:**
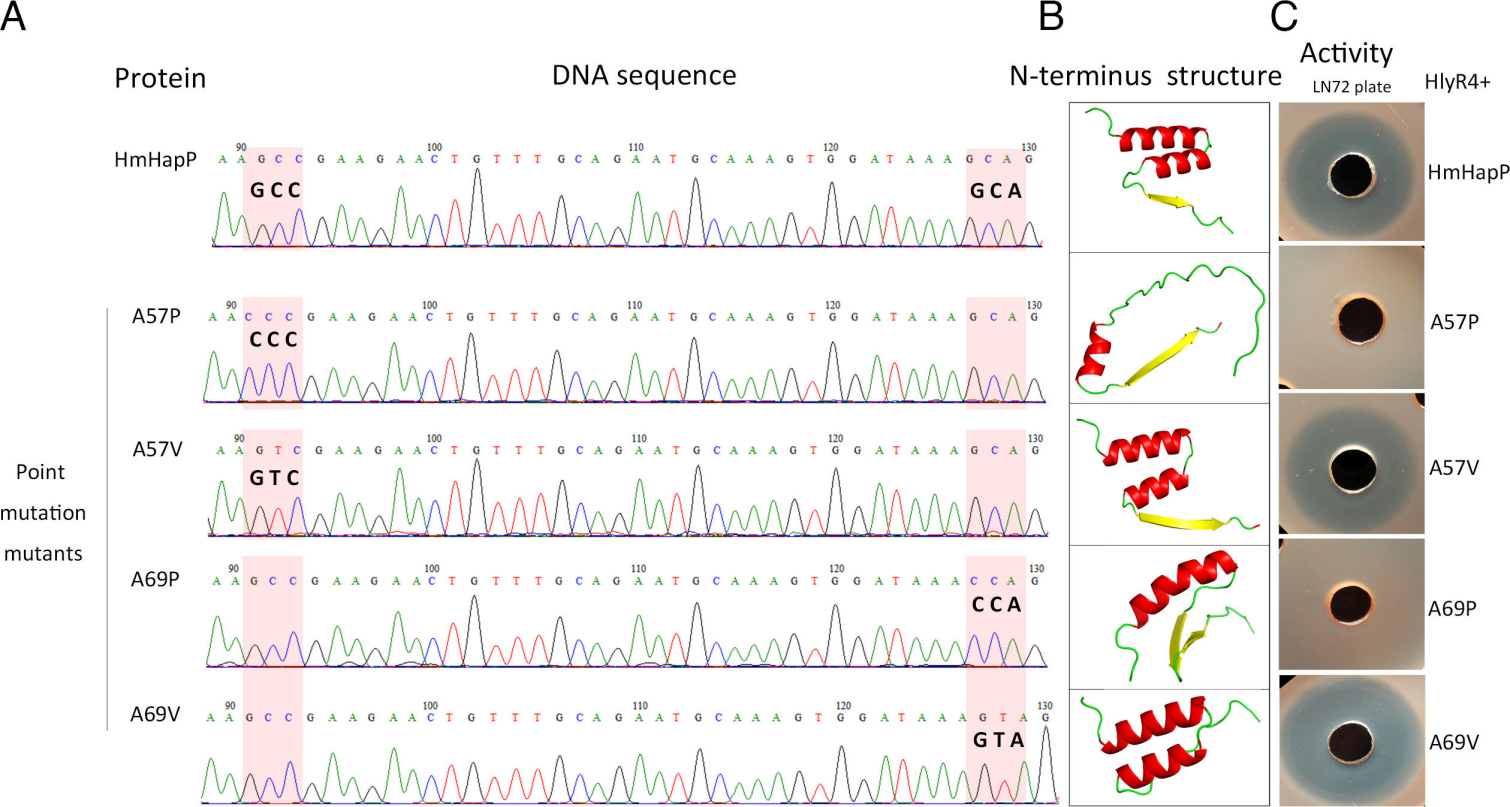
Correlation of secondary structure of the N-terminus of the HmHapP and its antagonistic activity. DNA sequencing was used to determine the point mutation mutants before protein expression. The nucleotides in the pink shadow were indicating the point mutation position (**A**). The secondary structure of the N-terminus (45 aa) of HmHapP and its point mutation mutants was predicted using AlphaFold3 (https://alphafold.com/) (**B**). Proteins A57P, A57V, A69P, and A69V, as well the HmHapP (wild type was taken as positive control), were cleaved by HlyR4, and then, the resulting mixtures were dropped into the holes punched on strain LN72 agar plate for inhibition activity detection after an incubation at room temperature of 1 h (**C**).

AlphaFold3 structural predictions indicated that the N-terminal 45-amino-acid region of HmHapP (HmHapP-N) contains two characteristic α-helices (Fig. 6B and 7C). To investigate whether these structural elements are essential for antimicrobial activity, we introduced proline—a known disruptor of α-helical conformation—at key positions within each helix. Alanine residues at positions 57 and 69 were separately mutated to proline (A57P and A69P) or valine (A57V and A69V) as structural controls (Fig. 9A). Secondary structure predictions confirmed that both A57P and A69P severely disrupted helix formation (Fig. 9B), and these mutants lost nearly all inhibitory activity (Fig. 9C). In contrast, the A57V and A69V mutants, which preserve α-helical structure, retained wild-type levels of antimicrobial function (Fig. 9C). These results demonstrate that the integrity of the α-helical domains in HmHapP-N is critical for its antagonistic activity.

### Fusion of HmHapP-N converts HlyR4 into an antimicrobial protease

**Fig 10:**
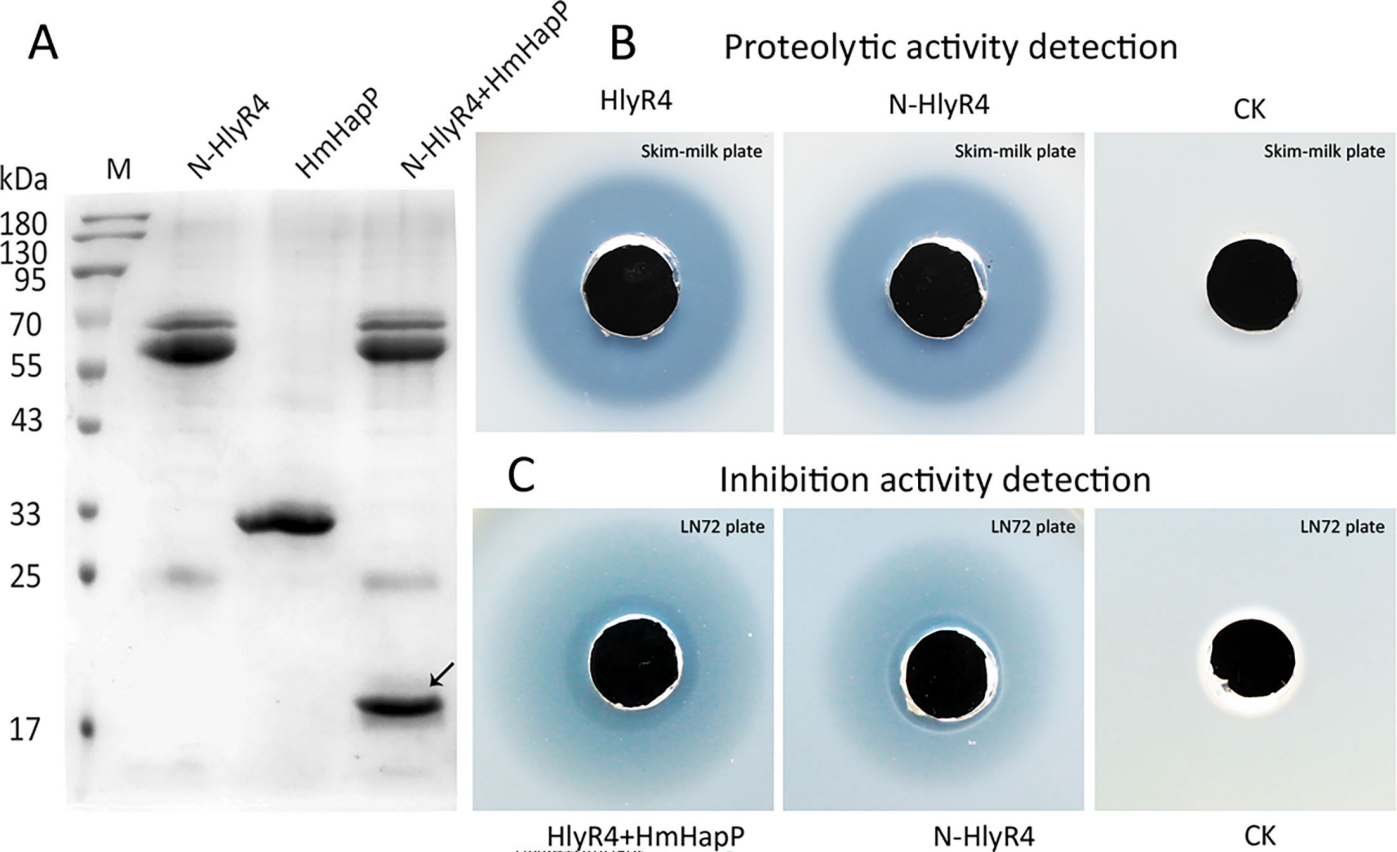
Proteolytic and inhibition activities of the fused protein N-HlyR4. The protein N-HlyR4 was constructed by fusing the N-terminal 45 aa with halolysin HlyR4 at the N-terminus. The N-HlyR4 can also cleave HmHapP into a N-terminus (45 aa) and a C-terminus (black arrow) (**A**). The N-HlyR4, like HlyR4, also shows a clear proteolytic activity on skim-milk plate (**B**). After refolding, the N-HlyR4 shows an obvious inhibition activity on the indicating agar plate with lawn cells of strain LN72 (**C**). CK, the refolding buffer (4.0 M NaCl, 50.0 mM Tris-HCl, 10.0 mM CaCl_2_ pH 8.0).

The 45-amino-acid peptide HmHapP-N exhibits potent antihaloarchaeal activity (Fig. 8 and 9), whereas the halolysin HlyR4 alone shows no intrinsic antimicrobial function (20). To explore whether the antimicrobial property of HmHapP-N could be transferred to HlyR4, we constructed a fusion protein, N-HlyR4, by linking HmHapP-N to the N-terminus of HlyR4 (Fig. S19A and B). The resulting fusion protein retained full proteolytic activity, cleaving HmHapP efficiently (Fig. 10A) and forming a clear hydrolysis halo on skim-milk agar plates (Fig. 10B). Refolding and maturation of N-HlyR4 followed a pattern similar to that of wild-type HlyR4, involving removal of the N-terminal propeptide to generate the mature enzyme (Fig. S19C and D).

Notably, the N-HlyR4 fusion also exhibited strong antihaloarchaeal activity (Fig. 10C), indicating that the HmHapP-N domain confers antimicrobial function without interfering with the protease’s structural folding, maturation, or catalytic activity. These results demonstrate that HmHapP-N functions as a modular antimicrobial peptide, capable of acting independently or endowing heterologous proteins with antihaloarchaeal activity when fused in a structurally permissive context.

### HlyR4 cleaves multiple precursor proteins to confer antagonistic activity

Having established HmHapP as a bona fide precursor protein activated by HlyR4 *in vitro*, we sought to determine whether additional proteins with similar functions exist *in vivo*. To this end, we constructed a *hapP* (*hfx_0892*) knockout mutant (EPSR∆hapP) in the parental strain EPSR using the plasmid pHFX-hapP-UP-DW (Fig. S20) via a pop-in/pop-out strategy (22). A complementation strain (EPSR∆hapP::hapP) was generated by introducing the *hapP* gene cloned into shuttle vector pWL502 (Fig. S21) back into the knockout mutant. Successful construction of the wild-type, knockout, and complementation strains was confirmed by PCR (Fig. S22).

**Fig 11:**
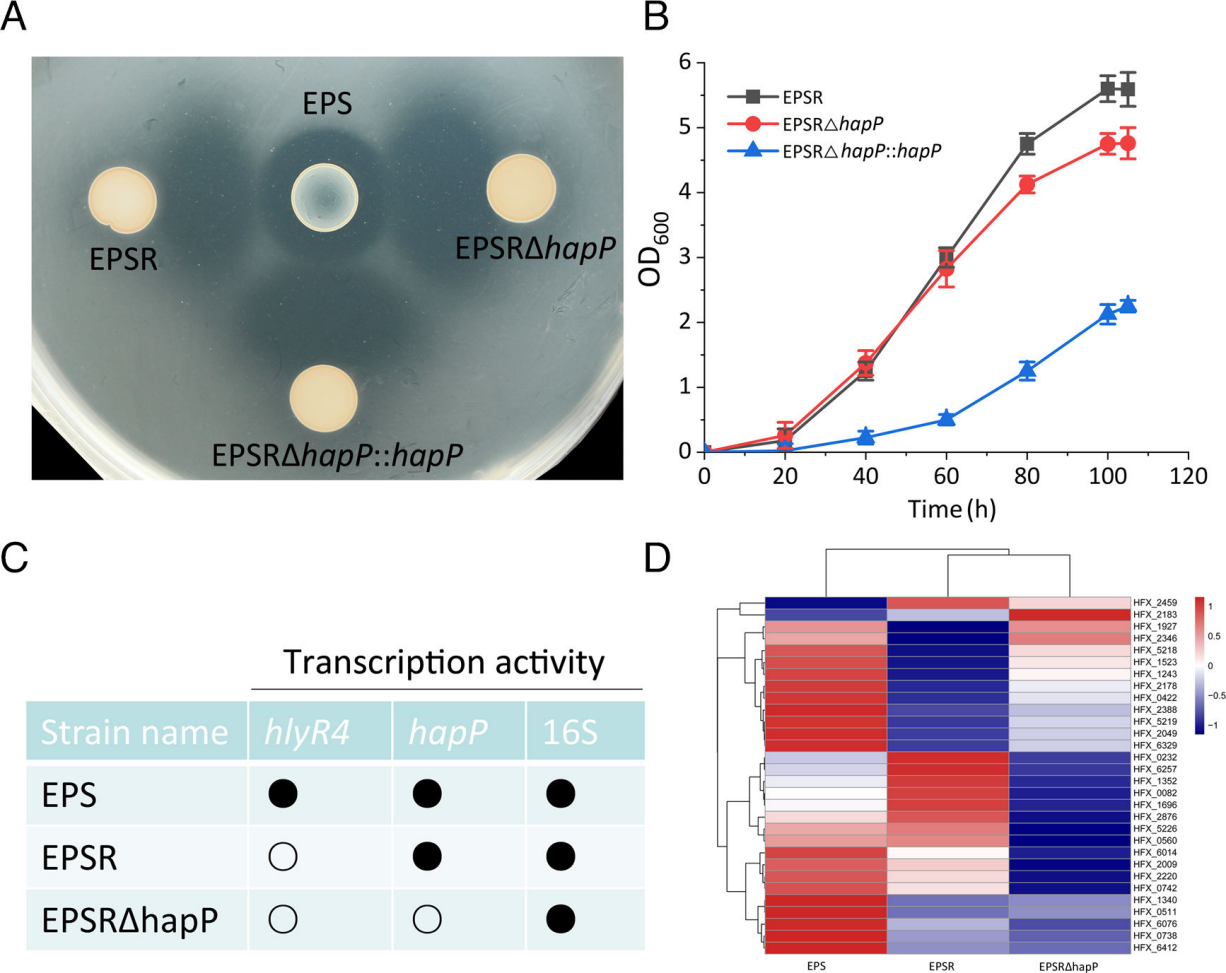
Impact of *hapP* on forming synergistic antagonism interaction, cell growth, and transcription profile. (**A**) The *hapP*-deficient mutant, EPSRΔ*hapP*, was generated using a pop-in/pop-out gene knockout strategy. The mutant was checked by PCR amplification with specific primers (Table 2). The *hapP*-complementary strain, EPSRΔ*hapP::hapP,* was constructed by transforming the recombinant plasmid pWL-hapP conceiving the *hapP* next to the *phaR* promoter into the EPSRΔ*hapP* strain. Strains EPS and EPSR were the same as these described in Fig. 1. Detection of synergistic antagonism was carried out on the LN72 plate. (**B**) Cell growth comparison of strains EPSR, EPSRΔ*hapP,* and EPSRΔ*hapP::hapP*. (**C**) Transcription activities of the *hlyR4*, *hapP,* and 16S rRNA gene (16S for short) in the cells of strains EPS, EPSR, and EPSRΔ*hapP*. (**D**) The 30 genes with the greatest variation of transcriptional level among strains EPS, EPSR, and EPSRΔ*hapP*. The variation of transcription was measured using log_2_FC; FC, fold change. The predicted functions of these 30 genes were listed in Table S2.

In paired-cultivation assays (Fig. S23), the *hapP* knockout strain retained the ability to form synergistic inhibition zones (Fig. 11A), indicating that other precursor proteins functionally analogous to HmHapP can be cleaved by halolysins to generate antimicrobial effectors. Deletion of *hapP* did not significantly affect growth (Fig. 11B), but RNA-seq analysis verified complete gene excision and revealed widespread transcriptional changes (Fig. 11C). Among the top 30 differentially expressed genes (Table S2), most—including *acnA*, *ftsZ*, *lonB*, *gvpA*, *gdhB*, and *phaP*—were markedly upregulated in the knockout strain (Fig. 11D; Table S2).

## DISCUSSION

In hypersaline environments, haloarchaea engage in ecological competition through the production of proteinaceous antagonists that inhibit closely related strains (23). While such interference competition has traditionally been attributed to halocins (24–26), the molecular basis of antagonism in model strains such as *Haloferax mediterranei* R4 (=ATCC 33500) has proven more complex. Although *halH4* was the first halocin gene cloned from this strain (27), its deletion did not abolish antimicrobial activity against *Halobacterium salinarium* NRC817 (26, 28), thereby prompting a search for additional antagonistic determinants.

Subsequently, genetic dissection revealed that the extracellular protease gene *hlyR4* is essential for strain R4’s inhibitory phenotype (19). In this system, strain R4 secretes both the precursor HalH4 and the extracellular serine protease HlyR4. Upon extracellular interaction, HlyR4 cleaves and activates HalH4, generating the antimicrobial C-terminal fragment CTH4 (20). This proteolytic activation mechanism parallels that of HalC8, which also releases a bioactive C-terminal fragment upon N-terminal cleavage, though its cognate protease remains unidentified (17, 29). However, not all halocin-like precursors follow this maturation pattern.

During the elucidation of the “synergistic antagonism” phenomenon (Fig. 1), we identified HFX_0892, a precursor protein activated within the synergistic antagonism zone (Fig. 3 to 6). Unlike HalH4 (20) and HalC8 (29), HFX_0892 releases an N-terminal bioactive fragment (Fig. 7), resembling the processing of HalS8 (17, 30). Structural and functional analyses confirmed that an N-terminal alpha-helix is essential for its antagonistic activity (Fig. 9). This mechanism mirrors trypsin-mediated activation of the crayfish antimicrobial peptide PcnAMP, which also liberates an N-terminal helical fragment active against *Aeromonas hydrophila* (31).

Beyond halocins, haloarchaea produce a range of antimicrobial agents beyond halocins, underscoring their potential as sources of novel bioactive compounds. Carotenoids from *Haloferax*, *Halogeometricum*, and *Haloarcula* exhibit activity against human and fish pathogens (12), whereas *Haloferax*-derived silver chloride nanoparticles show broad antimicrobial properties (32). Additionally, contractile injection systems (CISs) in *Halogeometricum borinquense* mediate interarchaeal antagonism through direct cell lysis (33). Recent genome mining efforts have further identified lanthipeptides such as archalan α and β from *Halorussus salinus* (11), and *in silico* screening of archaeal proteomes has predicted thousands of potential antimicrobial “archaeasins,” 93% of which demonstrated *in vitro* activity (34).

Notably, some haloarchaeal antimicrobials target bacteria. Peptidoglycan hydrolases (PGHs) encoded in genomes of strains such as *Halogranum salarium* B-1 lyse halophilic bacteria via peptidoglycan cleavage (13), despite archaea lacking this polymer. This function is analogous to the metalloprotease pseudoalterin from *Pseudoalteromonas* sp. CF6-2, which kills gram-positive bacteria by hydrolyzing peptidoglycan (35).

Thus, haloarchaeal antimicrobial strategies can be broadly categorized into halocins, which primarily target archaea, and PGHs, which act against bacteria. While over 17 halocins have been reported (36), the recent discovery of PGHs highlights the functional diversity of haloarchaeal antagonistic factors (13). In this work, we discover the “synergistic antagonism” as a distinct interaction mode in which an extracellular protease (e.g., HlyR4) from one strain activates a precursor protein (e.g., HFX_0892) from another, leading to specific cleavage and effector (0892N) release. Although halophilic bacterial proteases can substitute for HlyR4 in this system, no native bacterial precursor has been identified. Future efforts will focus on discovering such precursors to expand the repertoire of antibacterial agents.

In summary, this study provides a framework for uncovering cryptic antimicrobial peptides in the largely unexplored functional "dark matter" of halophiles, thereby expanding our understanding of complex microbial interactions in extreme environments.

## MATERIALS AND METHODS

### Microbial strains and cultivation conditions

Haloarchaeal strains, along with halophilic and salt-tolerant bacteria, were cultivated in JCM 168 medium (https://jcm.brc.riken.jp/en/). For the maintenance and propagation of the wild-type *Haloferax mediterranei* EPS and its genetically modified derivatives—including the extracellular protease-deficient mutant EPSR (∆*hlyR4*), the double knockout EPSR∆*hapP*, and the complementation strain EPSR∆*hapP::hapP* (Table 1)—the medium was supplemented with uracil (Sigma-Aldrich, Shanghai, China) at a final concentration of 50 µg·mL^−1^. In this study, the gene *hfx_0892* was designated *hapP*, and its encoded protein was named HmHapP (*Haloferax mediterranei*-derived haloarchaeal antagonistic precursor protein). Haloarchaeal cultures were incubated at 42°C with shaking at 180 rpm in liquid medium, and cell growth was monitored by measuring optical density at 600 nm (OD_600_).

**TABLE 1:** Strains and plasmids

Name	Description	Source or reference
Strains		
EPS	*Haloferax mediterranei* ATCC33500; *pyrF^-^*; *eps^-^*	(37)
EPSR	Strain EPS with a deletion of the *hlyR4*; *hlyR4^-^*	(19)
EPSRΔ*hapP*	Strain EPSR with a deletion of the *hapP*; *hapP^-^*	This study
EPSRΔ*hapP*::*hapP*	Strain EPSRΔ*hapP* harboring the complement plasmid pWL-hapP; *hapP^+^*	This study
*Halorubrum* sp. F4	Wild type haloarchaeal strain used for lawn cells in inhibition activity detection	(19)
*Halorubrum* sp. LN72	Wild type haloarchaeal strain used for lawn cells in inhibition activity detection	(19)
*Haloferax* sp. Q22	Wild type haloarchaeal strain used for lawn cells in inhibition activity detection	(19)
*Escherichia coli* JM109	Widely used strain for molecular cloning, *recA1*, *endA1*, *gyrA96L, thi*^-^, *hsdR17*, *supE44*, *relA1*	Novagen, USA
*E. coli* JM110	The *dam-* and *dcm-* of *E. coli* JM 109	TaKaRa, Japan
*E. coli* BL21 (DE3)	F^-^*omp*T^-^*hsd*SB (*rB*^-^*m*B^-^) *gal*^-^ *dcm*^-^(DE3)	Novagen, USA
Plasmids		
pET-23b (+)	Expression vector in *E. coli*; 3,665 bp	Novagen, USA
pET23b-R4	The *hlyR4* (*HFX_2419*) inserted into the vector pET-23b(+) at *Nde*I and *Eco*RI site, Amp^R^; ~5.2 kb	(19)
pET23b-hly65	Gene synthesis after codon usage optimization; the *hly65* (PP992913; 1,581 bp) from *Halorubellus* sp. PRR65 was optimized and inserted into the pET23b(+) at *Nde*I and *Hin*dIII site, Amp^R^; ~5.2 kb	(38)
pET23b-sptA	Gene synthesis after codon usage optimization; the *sptA* from *Natrinema* sp. J7 (1,695 bp) was optimized and inserted into the pET23b(+) at *Nde*I and *Hin*dIII site, Amp^R^; ~5.3 kb	This study
pET23b-hly_Hap_	The *hly*_Hap_ (1, 203 bp) from *Haladaptatus* sp. DYF46 was inserted into the pET23b(+) at *Nde*I and *Hin*dIII site, Amp^R^; ~4.8 kb	(39)
pET23b-hapP	Generated by gene synthesis after codon usage optimization according to *E. coli*; the optimized *hapP* (HFX_0892) from *Hfx. mediterranei* strain EPS inserted into the pET23b(+) at *Nde*I and *Hin*dIII site, Amp^R^; ~4.4 kb	This study
pET23b-hapP-DU30	Plasmid pET-23b(+) conceiving the *hapP* with a deletion of the fragment encoding the N-terminal 30 aa; Amp^R^; ~4.3 kb	This study
pET23b-hapP-DU50	Plasmid pET-23b(+) conceiving the *hapP* with a deletion of the fragment encoding the N-terminal 50 aa; Amp^R^; ~4.2 kb	This study
pET23b-hapP-DD50	Plasmid pET-23b(+) conceiving the *hapP* with a deletion of the fragment encoding the C-terminal 50 aa; Amp^R^; ~4.2 kb	This study
pET23b-hapP-C	Plasmid pET-23b(+) conceiving the DNA fragment encoding the C-terminal 190 aa of HmHapP at *Nde*I and *Hin*dIII site, Amp^R^; ~4.0 kb	This study
pET23b-hapP-N	Plasmid pET-23b(+) conceiving the DNA fragment encoding the N-terminal 45 aa of HmHapP at *Nde*I and *Hin*dIII site, Amp^R^; ~3.7 kb	This study
pET23b-A57V	Plasmid pET-23b(+) conceiving the *hapP* with an amino acid mutation at position 57 (A57V); Amp^R^; ~4.4 kb	This study
pET23b-A57P	Plasmid pET-23b(+) conceiving the *hapP* with an amino acid mutation at position 57 (A57P); Amp^R^; ~4.4 kb	This study
pET23b-A69V	Plasmid pET-23b(+) conceiving the *hapP* with an amino acid mutation at position 69 (A69V); Amp^R^; ~4.4 kb	This study
pET23b-A69P	Plasmid pET-23b(+) conceiving the *hapP* with an amino acid mutation at position 69 (A69P); Amp^R^; ~4.4 kb	This study
pET23b-D154A	Plasmid pET-23b(+) conceiving the *hlyR4* with an amino acid mutation at position 154 (D154A), Amp^R^; ~5.2 kb	This study
pET23b-H193R	Plasmid pET-23b(+) conceiving the *hlyR4* with an amino acid mutation at position 193 (H193R), Amp^R^; ~5.2 kb	This study
pET23b-S347A	Plasmid pET-23b(+) conceiving the *hlyR4* with an amino acid mutation at position 347 (S347A), Amp^R^; ~5.2 kb	This study
pET23b-W242C	Plasmid pET-23b(+) conceiving the *hlyR4* with an amino acid mutation at position 242 (W242C), Amp^R^; ~5.2 kb	This study
pET23b-Q274H	Plasmid pET-23b(+) conceiving the *hlyR4* with an amino acid mutation at position 274 (Q274H), Amp^R^; ~5.2 kb	This study
pET23b-E417D	Plasmid pET-23b(+) conceiving the *hlyR4* with an amino acid mutation at position 417 (E417D), Amp^R^; ~5.2 kb	This study
pET23b-T419N	Plasmid pET-23b(+) conceiving the *hlyR4* with an amino acid mutation at position 419 (T419N), Amp^R^; ~5.2 kb	This study
pET23b-N-hlyR4	The DNA sequence encoding the HmHapP-N linked to the 5’ terminal of the hlyR4 without signal peptide encoding sequence resulting in the *N-hlyR4*; the DNA fragment *N-hlyR4* inserted into the pET23b(+) at *Nde*I and *Hin*dIII site, Amp^R^; ~5.4 kb	This study
pHFX	A suicide plasmid in haloarchaea and can replicate in *E. coli*, Amp^R^, *pyrF^+^*; ~4.0 kb	(22)
pHFX-hapP-UP-DW	A DNA fragment consisting of a 543 bp up-stream and 537 bp down-stream DNA sequence of the *hapP* inserted into pHFX at the *Kpn*I site, Amp^R^; *pyrF^+^*; ~5.0 kb	This study
pWL502	Shuttle vector between haloarchaea and *E. coli*, Amp^R^, *pyrF^+^*; ~7.9 kb	(22)
pWL-hapP	Plasmid pWL502 conceiving the *hapP* along with its native promoter sequence (a 100 bp DNA sequence up-stream of the *hapP*), Amp^R^; *pyrF^+^*; ~8.7 kb	This study

For cocultivation systems involving both haloarchaea and bacteria, the temperature was maintained at 37°C to accommodate the growth requirements of both domains. Solid media were prepared by adding 1.5% (wt/vol) agar (Thermo Fisher Scientific, Waltham, MA, USA) prior to autoclaving at 121°C for 20 min.

*E. coli* strains JM109 and BL21(DE3), used for plasmid propagation and recombinant protein expression, were grown in Luria–Bertani medium (40). Where appropriate, ampicillin (Sigma-Aldrich, Shanghai, China) was added to a final concentration of 100 µg·mL^−1^.

### Screening for synergistic antagonism

The detection of synergistic antagonism employed a tripartite cocultivation system (Fig. S23). This system consisted of (i) an extracellular protease-producing strain (e.g., *Haloferax mediterranei* EPS), (ii) a putative precursor protein-producing strain (e.g., the protease-deficient mutant EPSR), and (iii) a lawn of cells sensitive to the activated effector molecules generated by protease-mediated cleavage of the precursor protein (e.g., *Halorubrum* sp. LN72 or *Haloferax* sp. Q22). The lawn strain served as a bioindicator for the presence of functional antimicrobial activity resulting from the synergistic interaction between strains A and B.

### Strains classification and deposition

Wild-type haloarchaeal strains served multiple experimental roles: as lawn cells for determining the antimicrobial spectrum (Table 3), and as potential substitutes for strains EPS or EPSR in screening for synergistic antagonism (Table 4). All strains used to replace strain EPS were confirmed extracellular protease producers. Taxonomic identification of these strains was performed using primers listed in Table 2.

**TABLE 2:** Primers used in this study^*a*^

Name	Sequence (5′−3′)	Description
F8	TTGATCCTGCCGGAGGCCATTG	For amplifying and sequencing the 16S rRNA gene of haloarchaea
R1462	ATCCAGCCGCAGATTCCCCTAC	
T7F	TAATACGACTCACTATAGGG	For DNA sequencing and detecting the fragment inserting into vector pET-23b(+)
T7R	TGCTAGTTATTGCTCAGCGG	
H4F	GCCATATGGCGACCGGCTCATCGGTTAC	For amplifying *halH4* (970 bp)
H4R	GCAAGCTTCTGTTTCCTACTCCGTTGTT	
R4F	GCCATATGGCAGGCACACCGAACTTCGA	For amplifying *hlyR4* (1,557 bp)
R4R	GCGAATTCTTCCTTTTCCGTGATGGTCA	
UPhapPF	GCAAGCTTCGACGGCGAATGACTGTCG	For amplification of 543 bp up-stream of the *hapP*
UPhapPR	TGAGTGGTTGAGTTGCAAGAGACGATTGGACTGAT	
DWhapPF	TCCAATCGTCTCTTGCAACTCAACCACTCACGC	For amplification of 537 bp down-stream of the *hapP*
DWhapPR	GCAAGCTTTGTCGGAGCTTCAAGCCGGG	
ComhapPF	GCGGTACCGTGACACTGACTAGTAGAGT	For amplification of the *hapP* and upstream 100 bp (1,040 bp)
ComhapPR	GCGGATCCTAGCGTGAGTGGTTGAGTTG	
hapPF	GCCATATGATGAAGATGTCAACCGTTAA	For amplification of the *hapP* without the signal peptide encoding sequence; 705 bp
hapPR	GCAAGCTTAGCGTCTTTCCCAGCAAAAC	
hapPDU30F	GCCATATGCAGGGTGGTGGCACCGTGTA	For amplification of the *hapP* without N-terminal 30 aa encoding sequence; 615 bp
hapPR	GCAAGCTTAGCGTCTTTCCCAGCAAAAC	
hapPDU50F	GCCATATGAGTGAAGCCAATGAAAATGA	For amplification of the *hapP* without N-terminal 50 aa encoding sequence; 555 bp
hapPR	GCAAGCTTAGCGTCTTTCCCAGCAAAA	
hapPF	GCCATATGATGAAGATGTCAACCGTTAA	For amplification of the *hapP* without C-terminal 50 aa encoding sequence; 555 bp
hapPDD50R	GCAAGCTTCGGCGGTTTGCTGCTGGCGG	
hapPCF	GCCATATGAGTGATTATAATCAGAGTGA	For amplification of the sequence encoding the *hapP* C-terminal 190 aa; 570 bp
hapPR	GCAAGCTTAGCGTCTTTCCCAGCAAAAC	
A57PF	GCTGAAAGAACCCGAAGAAC	Reverse PCR primers for construction mutant A57P
A57PR	GTTCTTCGGGTTCTTTCAGC	
A57VF	GCTGAAAGAAGTCGAAGAAC	Reverse PCR primers for construction mutant A57V
A57VR	GTTCTTCGACTTCTTTCAGC	
A69PF	GTGGATAAACCAGTTAGCGCC	Reverse PCR primers for construction mutant A69P
A69PR	GGCGCTAACTGGTTTATCCAC	
A69VF	GTGGATAAAGTAGTTAGCGC	Reverse PCR primers for construction mutant A69V
A69VR	GCGCTAACTACTTTATCCAC	
hapPF	GCCATATGATGAAGATGTCAACCGTTAA	For amplifying the 135 bp of the 5’-terminus of the *hapP*
N-HAPR1	GTTCGGTGTGCCTGCTTTGCTCAGTTCGCT	
N-HlyR4F2	AGCGAACTGAGCAAAGCAGGCACACCGAAC	For amplifying the *hlyR4* without the signal peptide encoding sequence
N-HlyR4R2	GCGGATCCTCCTTTTCCGTGAT	
W242CF	GCTATCGAGTGCGCCGCAGAC	Reverse PCR primers for construction mutant W242C
W242CR	GTCTGCGGCGCACTCGATAGC	
Q274HF	GCGACGCAGCACGGTTCGCTC	Reverse PCR primers for construction mutant Q274H
Q274HR	GAGCGAACCGTGCTGCGTCGC	
E417DF	GGTTCGAAGGATACGACCTAC	Reverse PCR primers for construction mutant E417D
E417DR	GTAGGTCGTATCCTTCGAACC	
T419NF	AAGGAAACGAACTACGATGGG	Reverse PCR primers for construction mutant T419N
T419NR	CCCATCGTAGTTCGTTTCCTT	

^*a*^ The restriction sites are underlined.

All strains were preserved in 25% (vol/vol) glycerol at –80°C. Selected lawn strains have been deposited in public culture collections, with *Halorubrum* sp. F4 accessible as CGMCC 1.16023 and NBRC 112865, and *Halorubrum* sp. LN72 as CGMCC 1.62755.

### Preparation of indicator plates and agar diffusion assay

Lawn cultures were prepared using strains harvested at mid-log phase (OD_600_= 1.0–1.5). For each indicator plate, 10 mL of cell culture was aseptically mixed with 100 mL of molten JCM 168 medium cooled to approximately 55°C after autoclaving. The mixture was gently swirled to ensure uniform cell distribution, and approximately 20 mL was poured into each sterile plastic Petri dish (ϕ = 9 cm). Once solidified, 6 mm diameter wells were punched into the agar for use in subsequent diffusion assays.

### Antimicrobial spectrum and synergistic assays

The antimicrobial spectrum was assessed using strains listed in Table 3 as lawn cells. For synergistic antagonism screening, 10 μL suspensions of strains EPS and EPSR were spotted onto indicator plates with their centers spaced 3 cm apart. To evaluate the effect of distance on inhibition zone formation, inter-colony distances were systematically varied from 2 to 6 cm. In some experiments, a narrow strip of agar was aseptically removed between inoculation points prior to cultivation to ensure interaction between diffusible compounds.

**TABLE 3:** Strains used for screening inhibition spectrum^*a*^

Type	Strain name	Taxonomic position	Accession no. of the 16S rRNA gene	Habitat	Inhibitory activity
Archaea	FL35	*Haloarcula japonica*	OM884235	Salted seaweed	+
	LN187 (19)	*Halorubrum californiense*	MN826837	Salt mine, China	-
	LN27 (19)	*Halorubrum aidingense*	MN829451	Salt mine, China	-
	ZY8	*Halorubrum lacusprofundi*	NR_165753	Salt mine, China	+
	LN72 (19)	*Halorubrum* sp.	MN829452	Salt mine, China	+
	F4 (19)	*Halorubrum* sp.	MG097853	Salt mine, China	+
	LN28	*Halorubrum lipolyticum*	OM884238	Salt mine, China	+
	LN39 (19)	*Halorubrum lipolyticum*	MH620371	Salt mine, China	-
	LN60 (19)	*Halorubrum aquaticum*	MN826834	Salt mine, China	+
	F18S	*Halorubrum coriense*	OM865838	Salt mine, China	+
	FL45 (19)	*Halolamina pelagica*	MN833413	Salted seaweed	+
	DYS4 (19)	*Haloparvum sedimenti*	NR_149276	Salt mine, China	+
	D90 (19)	*Halobaculum magnesiiphilum*	KX376701	Salt mine, China	-
	D93	*Halobaculum magnesiiphilum*	KX376702	Salt mine, China	-
	LN185	*Halopenitus malekzadehii*	OM884247	Salt mine, China	-
	GSM4	*Halobellus rufus*	OM865837	Salt mine, China	+
	ZY21	*Halobellus rufus*	NR_165787	Salt mine, China	+
	LR2	*Natrialba asiatica*	OM865836	Salt mine, China	-
	Y13	*Haloferax denitrificans*	OM865835	Salt mine, China	-
	LN10 (19)	*Natrinema pellirubrum*	MN826830	Salt mine, China	-
	FL144	*Salinarchaeum laminariae*	OM884256	Salted seaweed, China	-
	FL84	*Salarchaeum japonicum*	OM884255	Salted seaweed, China	-
Bacteria	LN118	*Halomonas gudaonensis*	OM884258	Salt mine, China	-
	FL7	*Halomonas halmophila*	OM884260	Salted seaweed, China	-
	LN97	*Halomonas gudaonensis*	OM884261	Salt mine, China	-
	FL1	*Halomonas halmophila*	OM884257	Salted seaweed, China	-
	168-18	*Salinicoccus salsiraiae*	OM884259	Salt mine, China	-
	LN88	*Pseudomonas halophila*	OM884262	Salt mine, China	-

^*a*^ Strains can be used as sensitive lawn cells in detection of synergistic antagonism activity (shaded in gray). +, positive; −, negative.

### Protein and peptide activity analysis

For refolded or cleaved protein samples, solutions (1.0 mg·mL^−1^) were loaded into 6-mm wells punched in indicator lawns. To quantify bactericidal activity, peptides HmHapP-N and HmHapP-C (500 μL of 1.0 mg·mL^−1^ in refolding buffer) were incubated with cell pellets of strains F4 and LN72 harvested from mid-log phase cultures (OD_600_ = 1.0–1.5). After 24 h at 4°C, serial dilutions (10^−1^ to 10^−8^) were prepared in JCM 168 medium and spotted (10 μL) onto agar plates to determine viable counts. Parallel samples of LN72 cells treated with HmHapP-N for 0, 6, 12, and 24 h were processed for scanning electron microscopy (see corresponding section).

### Isolation and identification of antagonistic proteins

To isolate proteins responsible for antagonistic activity, strains EPS and EPSR (15 μL, OD_600_= 2.0) were inoculated simultaneously onto paired agar plates: an LN72 indicator lawn and a blank JCM 168 plate without sensitive cells, with inoculation points spaced 3 cm apart. Following 2 weeks of incubation, the FSIZ adjacent to the EPSR colony on the LN72 plate served as a spatial reference for protein recovery. Agar from the corresponding region on the blank plate was excised and diced into ∼0.5 cm^3^ cubes.

Protein extraction was performed using a modified DNA gel extraction column, wherein the original membrane was replaced with sterile absorbent cotton. The agar fragments were loaded into the column, and interstitial fluid containing secreted proteins was collected by centrifugation at 5,000 × *g* for 15 min. The eluate was concentrated via ultrafiltration (as described in “Protein refolding and concentration,” below), and proteins were precipitated with 40% (wt/vol) trichloroacetic acid (TCA). Precipitated proteins were separated by SDS-PAGE, and bands of interest were excised for in-gel tryptic digestion (Thermo Fisher Scientific, Waltham, MA, USA) prior to mass spectrometric identification (see “Mass spectrometry identification,” below).

### Proteolytic activity assay

Skim-milk agar plates were prepared by adding heat-treated (30 min in boiling water) 10% (wt/vol) skim milk (Gibco, New York, USA) to autoclaved JCM medium cooled to approximately 60°C. After thorough mixing, 20 mL of the mixture was poured into each Petri dish and allowed to solidify.

Proteolytic activity was assessed using two approaches: direct inoculation of cell suspensions onto the agar surface, or application of protein solutions into 6-mm wells punched in the skim-milk plates. Protease activity was indicated by the formation of clear hydrolysis zones around inoculation points or within wells following incubation.

### Functional screening for synergistic antagonism

To evaluate the role of extracellular proteases in synergistic antagonism, we performed strain substitution assays using protease-producing haloarchaea and halophilic/salt-tolerant bacteria (Table 4) as functional replacements for strain EPS. Concurrently, we screened a collection of laboratory-preserved haloarchaeal strains (Table 4) to identify candidates capable of substituting for strain EPSR as precursor protein producers.

**TABLE 4:** Strains used for replacement of strain EPS or strain EPSR in formation of synergetic antagonism^*a*^

Strain name	Taxonomic position	Accession no. of the 16S rRNA gene	Habitat	Synergetic antagonism activity	
				Replacing EPS	Replacing EPSR
J7	*Natrialba* sp.	MN826722	Natural salt, Bolivia	+	-
J9	*Natrialba* sp.	OM884248	Natural salt, Bolivia	-	-
J16	*Halorubrum* sp.	OM884240	Natural salt, Bolivia	-	-
J26	*Haloarcula* sp.	OM884236	Natural salt, Bolivia	-	-
J68	*Haloterrigena* sp.	MN826763	Natural salt, Bolivia	-	+
J81	*Halococcus* sp.	MH188862	Natural salt, Bolivia	-	-
J83	*Natrinema* sp.	OM884250	Natural salt, Bolivia	-	-
J162	*Halococcus* sp.	MN826762	Natural salt, Bolivia	+	-
J510	*Halococcus* sp.	MN836727	Natural salt, Bolivia	+	-
LN60	*Halorubrum* sp.	MN826834	Salt mine, China	-	-
LN68	*Pseudomonas* sp.	OM884264	Salt mine, China	-	-
LN54	*Natronoarchaeum* sp.	OM884254	Salt mine, China	-	+
AD209	*Saccharomonospora* sp.	OR994170	Saline-alkali soil, China	+	-
LN124	*Pseudomonas* sp.	OM884263	Salt mine, China	+	-
FL17	*Halolamina* sp.	OM884244	Salted seaweed, China	-	-
FL26	*Halobacterium* sp.	OM884252	Salted seaweed, China	-	-
FL66	*Haloarcula* sp.	OM884237	Salted seaweed, China	-	-
FL93	*Halohasta* sp.	MN833416	Salted seaweed, China	-	-
FL126	*Halobacterium* sp.	OM884252	Salted seaweed, China	-	-
LN16	*Haloterrigena* sp.	MN826832	Salt mine, China	-	+
FL176	*Haloarchaeobius* sp.	MW290930	Salted seaweed, China	+	-
FL187	*Halohasta* sp.	OM884251	Salted seaweed, China	-	-
FL23	*Halorubrum* sp.	MT573937	Salted seaweed, China	-	+
GM76	*Halobacillus* sp.	PV162520	Saline-alkali soil, China	+	-

^*a*^ +, positive result which means the strain can replace EPS or EPSR in generating synergetic antagonism activity (shaded in gray); -, negative result.

To further validate ecological relevance, we tested whether wild-type strains originating from the same habitat could recapitulate the synergistic interaction. Specifically, the co-isolated strains *Natrialba* sp. J7 and *Haloterrigena* sp. J68 were paired as functional substitutes for EPS and EPSR, respectively.

### Codon optimization and plasmid construction

To enhance heterologous expression in *E. coli*, the codon usage of haloarchaeal genes *hapP*, *hly65*, and *sptA*, as well as DNA fragments encoding HmHapP-N (N-terminal 45 aa) and HmHapP-C (C-terminal 190 aa), was optimized and synthesized by General Biol (Anhui, China). All sequences are listed in Table S1.

The synthesized fragments were cloned into the expression vector pET-23b(+) (Novagen, USA) using *Nde*I and *Hin*dIII restriction sites (New England Biolabs, USA). All constructs were verified by DNA sequencing prior to protein expression.

All primers used in this study are listed in Table 2. Gene cloning and plasmid propagation were performed in *E. coli* JM109, whereas protein expression was carried out in *E. coli* BL21(DE3). Gene fragments were amplified using KOD One PCR Master Mix (TOYOBO, Shanghai, China), purified with the AxyPrep DNA Gel Recovery Kit (AXYGEN, Hangzhou, China) following electrophoresis and assembled using restriction enzymes and T4 DNA ligase from New England Biolabs (Massachusetts, USA).

The *hlyR4* gene (HFX_2419) was amplified from the EPS genomic DNA and cloned into pET-23b(+) via *Nde*I and *Eco*RI sites, yielding plasmid pET23b-R4. Truncated mutants of HmHapP were amplified using the synthesized pET23b-hapP plasmid as template. All synthetic constructs, including point mutants and truncations, underwent codon optimization for enhanced expression in *E. coli* (Table 1). Plasmid pET23b-hlyHap was kindly provided by Dr. Hou (39).

For the construction of the fusion plasmid pET23b-N-hlyR4, two PCR fragments were generated: the 135-bp 5′-terminal region of *hapP* (Up fragment) and *hlyR4* lacking its signal peptide sequence (Down fragment). These fragments were fused by overlap extension PCR using primers hapPF and N-HlyR4R2. The resulting N-hlyR4 fusion fragment was cloned into pET-23b(+) using *Nde*I and *Hin*dIII sites through conventional restriction-ligation and transformation.

### Site-directed mutagenesis

Point mutations in HmHapP (A57P, A57V, A69P, and A69V) and HlyR4 (D154A, H193R, S347A, W242C, Q274H, E417D, and T419N) were introduced using inverse PCR with plasmids pET23b-hapP and pET23b-R4 as templates, respectively. Primer sequences are provided in Table 2.

The PCR products were purified, treated with *Dpn*I at 37°C for 15 min to remove template DNA, and heat-inactivated at 80°C for 20 min. The resulting plasmids were transformed into *E. coli* JM109. Positive clones were screened by PCR using 2× Hieff Ultra-Rapid II HotStart PCR Master Mix (Yeasen, Shanghai, China).

### Recombinant protein expression and purification

Recombinant protein expression in *E. coli* BL21(DE3) was induced with 0.6 mM isopropyl-β-d-thiogalactoside (IPTG) when cultures reached OD_600_= 0.4–0.8. After 4 h of post-induction incubation, the cells were harvested by centrifugation (13,400 × *g*, 3 min, 4 °C). Cell pellets were resuspended in lysis buffer (8 M urea, 50 mM Tris-HCl, 10 mM CaCl_2_, pH 8.0) and disrupted by ultrasonication on ice (20 min total, 5 s pulse/5 s pause intervals) using a SM-1000D sonicator (Shunma Tech, Nanjing, China).

All proteins expressed from the pET-23b(+) vector contained a C-terminal 6×His tag for purification. Clarified lysates were filtered through 0.22 μm membranes (Millex-GP, Merck, Germany) and applied to Ni-NTA Sefinose Resin (Sangon Biotech, Shanghai, China). The column was washed with four column volumes of wash buffer (8 M urea, 50 mM Tris-HCl, 10 mM CaCl₂, 30 mM imidazole, pH 8.0), and proteins were eluted with two column volumes of elution buffer (8 M urea, 50 mM Tris-HCl, 10 mM CaCl₂, 200 mM imidazole, pH 8.0) (41). Elution fractions were analyzed by SDS-PAGE to confirm purity and integrity.

### Protein refolding and concentration

To obtain active proteins from heterologous expression in *E. coli*, elution fractions containing the target protein were pooled and treated with 10 mM dithiothreitol (DTT; Biofroxx, Guangzhou, China) for 1 h at room temperature. The solution was then diluted 10-fold into refolding buffer (4.0 M NaCl, 50 mM Tris-HCl, 10 mM CaCl_2_, pH 8.0) and incubated at 37°C for 12 h. Refolded proteins were concentrated >10 fold using 3 kDa molecular weight cut-off (MWCO) ultrafiltration devices (PALL, Beijing, China) at 5,000 × *g* and 4°C. Protein concentration was determined with an ultramicro UV-visible spectrophotometer (ND-100, MIULAB, Hangzhou, China).

For the small peptide HmHapP-N, concentration was achieved by placing the solution in a 1 kDa MWCO dialysis membrane (Solarbio, Beijing, China) and embedding it in polyethylene glycol 8000 (PEG8000; Biosharp, Hefei, China) overnight at 4°C.

### Enzyme kinetics and activity assays

The kinetic parameters of HlyR4 were determined using an azocaseinolytic assay as previously described (38). Prior to kinetic analysis, the optimal temperature, NaCl concentration, and pH for enzymatic activity were established. The effects of organic reagents (10 mM PMSF, EDTA, and DTT) and inorganic ions (5 mM Fe^2+^, K^+^, Ca^2+^, Mn^2+^, Zn^2+^, Mg^2+^, and Cu^2+^) on enzyme activity were assessed using a microplate reader (TECAN, Shanghai, China).

Control reactions were prepared by adding trichloroacetic acid (TCA) to the substrate prior to enzyme addition. One unit (U) of azocaseinolytic activity was defined as the amount of enzyme required to increase OD_440_ by 0.01 per minute under standard assay conditions. Specific activity (U·μg⁻¹) was calculated as ΔOD_440_/(enzyme amount in μg)/0.01/60 (42, 43). All measurements were performed in triplicate.

### Protein cleavage assays

Proteolytic activity of S8-family halolysins (HlyR4, Hly65, HlyHap, and SptA) and their variants were assessed using HmHapP (HFX_0892) and its derivatives—including truncation mutants (DU30, DU50, and DD50) and point mutants (A57P, A57V, A69P, and A69V)—as substrates. Reactions contained 200 μL substrate (1.0 mg·mL⁻¹) and 50 μL protease (1.0 mg·mL⁻¹) in refolding buffer (4.0 M NaCl, 50 mM Tris-HCl, 10 mM CaCl_2_, pH 8.0) and were incubated at 37°C.

To monitor cleavage kinetics, time-course experiments were performed using 0.1 mg·mL⁻¹ HlyR4 with incubation times ranging from 0 to 30 min. Reactions were terminated by adding 250 μL of 40% (wt/vol) trichloroacetic acid (TCA); a zero-time control was prepared by adding TCA prior to protease. Precipitated proteins were collected by centrifugation (13,400 × *g*, 3 min), washed twice with acetone, air-dried, and analyzed by SDS-PAGE.

For routine cleavage validation, a standard 20-min incubation was employed.

### Protein complex structure prediction

Protein-protein complex structures were predicted using AlphaFold-Multimer (v2.3.0) (44). Multiple sequence alignments were generated via the ColabFold pipeline (v1.5.5) (45), with the max_multimer_length parameter adjusted to the total length of each complex. For each complex, five models were generated using the AlphaFold2-multimer-v2 preset and refined through three recycling iterations.

The final model was selected based on the highest predicted Local Distance Difference Test (pLDDT) score and the lowest Predicted Aligned Error (PAE) at the interaction interface. Residues with pLDDT>70 and inter-chain PAE<5 Å were classified as high-confidence interaction sites (46). Structural visualization of the HmHapP–HlyR4 complex was performed using LigPlus (47).

### Mass spectrometry identification

Protein bands of interest were excised from Coomassie Blue-stained SDS-PAGE gels and subjected to in-gel digestion with trypsin (Thermo Scientific Pierce, Shanghai, China). Mass spectrometric analysis was performed by the Public Service Platform at the Institute of Microbiology, Chinese Academy of Sciences, following the established protocol described by Chen et al. (20).

### Scanning electron microscopy

Haloarchaeal cells were fixed in a solution containing 2% (vol/vol) glutaraldehyde (Sigma-Aldrich, Shanghai, China) and 10% (wt/vol) NaCl overnight at 4°C. Fixed samples were then processed through standard dehydration and gold-sputtering procedures before examination under a HITACHI SU8010 scanning electron microscope (Japan), following the methodology outlined by Muller et al. (48).

### Protein structure analysis

Three-dimensional structures of HmHapP, HlyR4, and their derivatives were predicted using AlphaFold 3.0 (https://alphafoldserver.com/) (46). Resulting PDB files were visualized and analyzed with PyMOL 3.1 (https://pymol.org/2/) (49).

### Gene knockout and complementation

A 1,080-bp DNA fragment containing 543 bp upstream and 537 bp downstream of *hapP* was constructed via traditional and overlap extension PCR. This fragment was cloned into the suicide vector pHFX at the KpnI site, yielding the knockout plasmid pHFX-hapP-UP-DW (Table 1). Following passage through *E. coli* JM110, the plasmid was transformed into strain EPSR, and the *hapP*-deficient mutant EPSR∆*hapP* was obtained using the pop-in/pop-out strategy described by Chen et al. (28).

For genetic complementation, the *hapP* gene with its native promoter was amplified and inserted into the shuttle vector pWL502²¹, generating plasmid pWL-hapP (Table 1). This plasmid was introduced into EPSR∆*hapP* via *E. coli* JM110, producing the complemented strain EPSR∆*hapP::hapP*. All primers used for plasmid construction are listed in Table 2.

### Transcriptomic analysis

Total RNA was extracted using the Trizol method (TIANGEN, Beijing, China). RNA integrity and purity were assessed with a NanoDrop One spectrophotometer (Thermo Fisher Scientific, USA) and an Agilent 4200 TapeStation (Agilent Technologies, USA). Ribosomal RNA was depleted from qualified samples using the Epicenter Ribo-Zero rRNA Removal Kit (Illumina, USA). Strand-specific cDNA libraries were prepared with the NEBNext Ultra II Directional RNA Library Prep Kit (New England Biolabs, USA) and sequenced on an Illumina NovaSeq platform (PE150 mode).

Raw sequencing reads were quality-filtered using fastp v0.23.4 (50), followed by the removal of ribosomal sequences through alignment to the NCBI RefSeq and Rfam databases with Bowtie v2.5.4 (51). Unmapped reads were retained for downstream analysis. Transcript abundance was quantified with RSEM v1.1.17 (52), and differentially expressed genes were identified using DESeq v2.10 (53) with thresholds of |log₂ (fold change)| > 1 and adjusted *P*-value < 0.01.

## Data Availability

Strains EPS and EPSR were derived from the model haloarchaeon *Haloferax mediterranei* ATCC 33500. *Halorubrum* sp. F4 (accession numbers: CGMCC 1.16023, NBRC 112865) and *Halorubrum* sp. LN72 (CGMCC 1.62755) were isolated and identified in this study and have been deposited in the China General Microbiological Culture Collection Center (CGMCC) and the NITE Biological Resource Center (NBRC). The 16S rRNA gene sequences of strains F4 and LN72 are available in GenBank under accession numbers MG097853.2 and MN829452.2, respectively. Additional 16S rRNA gene sequences of strains used for antimicrobial spectrum screening are provided in Table 3, and those used in synergistic antagonism substitution assays are listed in Table 4. Transcriptomic data generated in this study are accessible under NCBI BioProject PRJNA1221529.

## SUPPLEMENTAL MATERIAL

Supplemental Material mbio.03405-25-s0001.pdf Figures S1 to S23; Tables S1 and S2.

ASM does not own the copyrights to Supplemental Material that may be linked to, or accessed through, an article. The authors have granted ASM a non-exclusive, world-wide license to publish the Supplemental Material files. Please contact the corresponding author directly for reuse.
