# Supplementary material for: A protease-precursor system drives synergistic antagonism in haloarchaea
Source: mBio. 2026 Jan 14;17(2):e03405-25. doi: 10.1128/mbio.03405-25 (PMC12893001; doi:10.1128/mbio.03405-25)
Supplement: Supplemental Material — Figures S1 to S23; Tables S1 and S2. [file mbio.03405-25-s0001.pdf]

Supplementary data

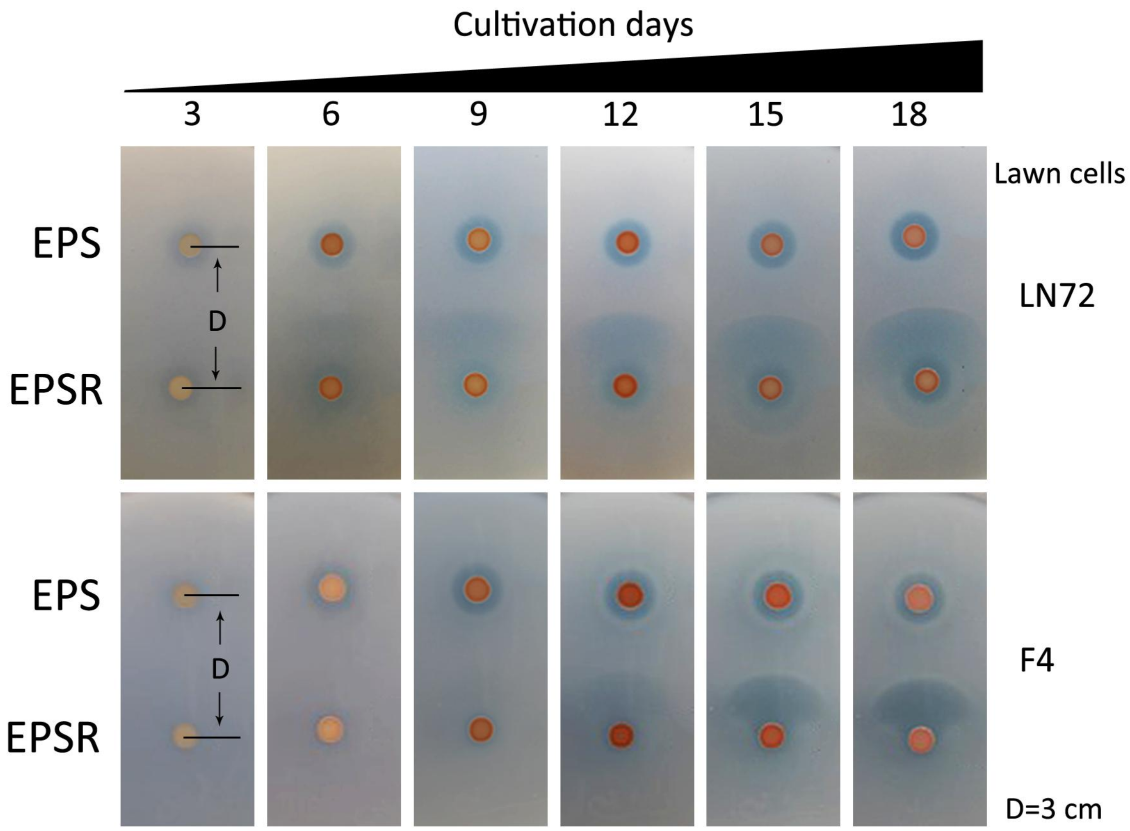

Fig. S1 The formation of synergistic antagonism between strains EPS and EPSR along with the time-course. EPS, *Hfx. mediterranei* strain EPS (EPS for short); EPSR, *Hfx. mediterranei* strain EPSR (EPSR for short), the halolysin deficient strain. D, the distance between two centers of colonies. Cells were cultivated on AS-168 medium supplemented with uracil (50 μg·mL^{-1}) for certain days at 42 °C. Lawn cells of strains LN72 and F4 belong to the species in genus *Halorubrum*.

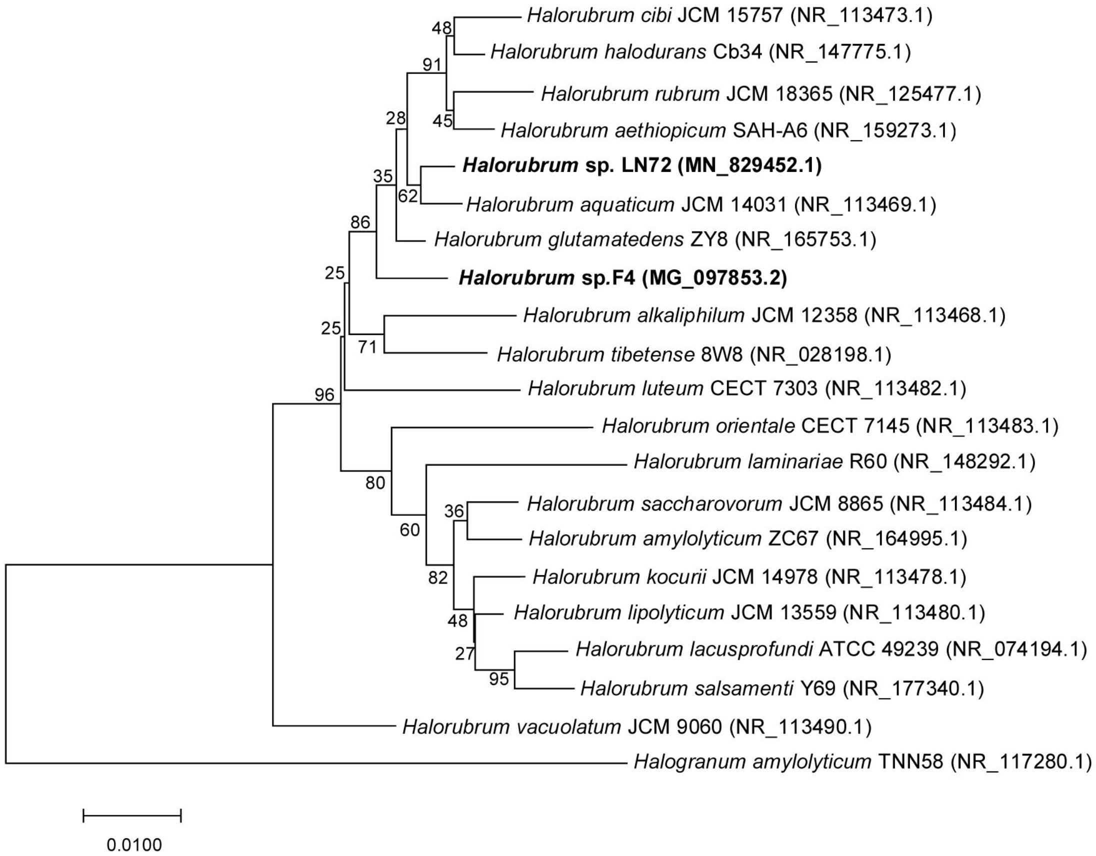

Fig. S2 Neighbour-joining phylogenetic tree based on the 16S rRNA gene sequences of strains F4 and LN72 in the genus *Halorubrum*. Percentage bootstrap values are shown at branch points. *Halogranum amylolyticum* TNN58 was used as an outgroup. Bar, 0.01 substitutions per nucleotide position.

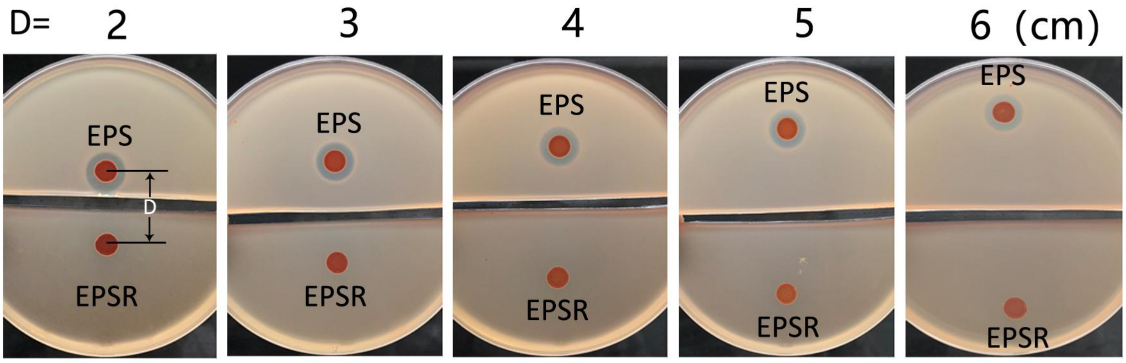

Figure S3. Segregating cultivation. Cutting out a strip of agar between strains EPS and EPSR insulates the substances exchange. Lawn cells: *Halorubrum* sp. F4. The distance between two colonies was set ranging from 2 to 6 cm. The agar plated was cultivated for 40 days on the AS-168 medium supplemented with uracil (50 $\mu g \cdot mL^{-1}$) at 42 °C.

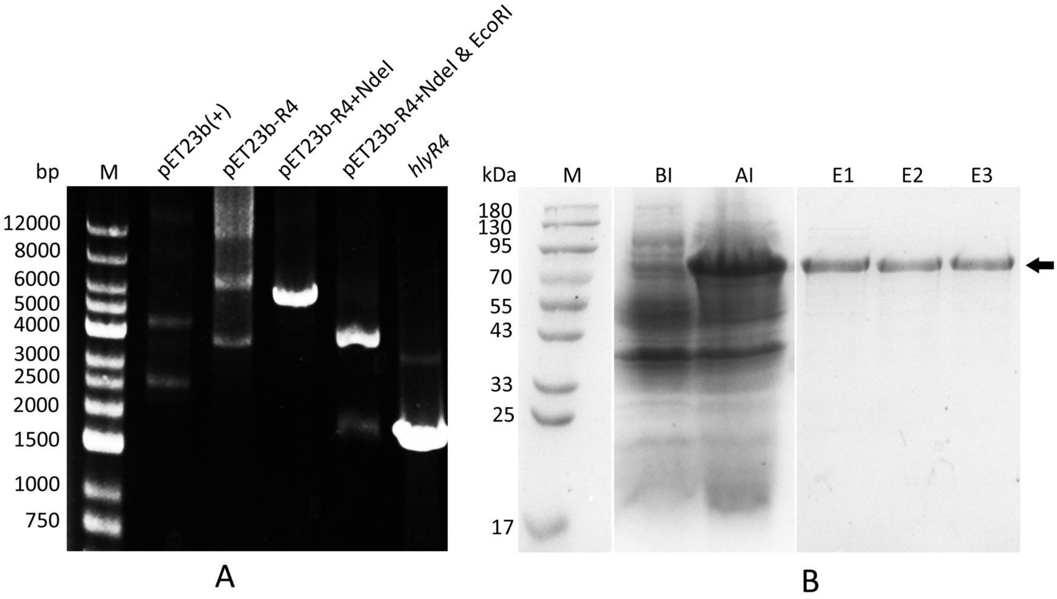

Fig. S4 Plasmid construction and gene expression of *hlyR4*. The *hlyR4* was inserted into the expression plasmid pET23b(+) resulting in the recombinant plasmid pET23b-R4. The plasmid pET23b-R4 was verified through single (*Nde*I) and double (*Nde*I and *Eco*RI) enzyme digestions (A) and DNA sequencing before protein expression. The plasmid pET23b-R4 was transformed into *E. coli* BL21 (DE3) for gene expression with IPTG induction. The effect of gene expression and protein purification with Ni-NTA affinity chromatography was checked by SDS-PAGE (B). BI, Before IPTG induction; AI, after IPTG induction; E1-E3, elutes; arrow shows the target products, HlyR4. The DNA ladder and protein molecular standard are shown on the left.

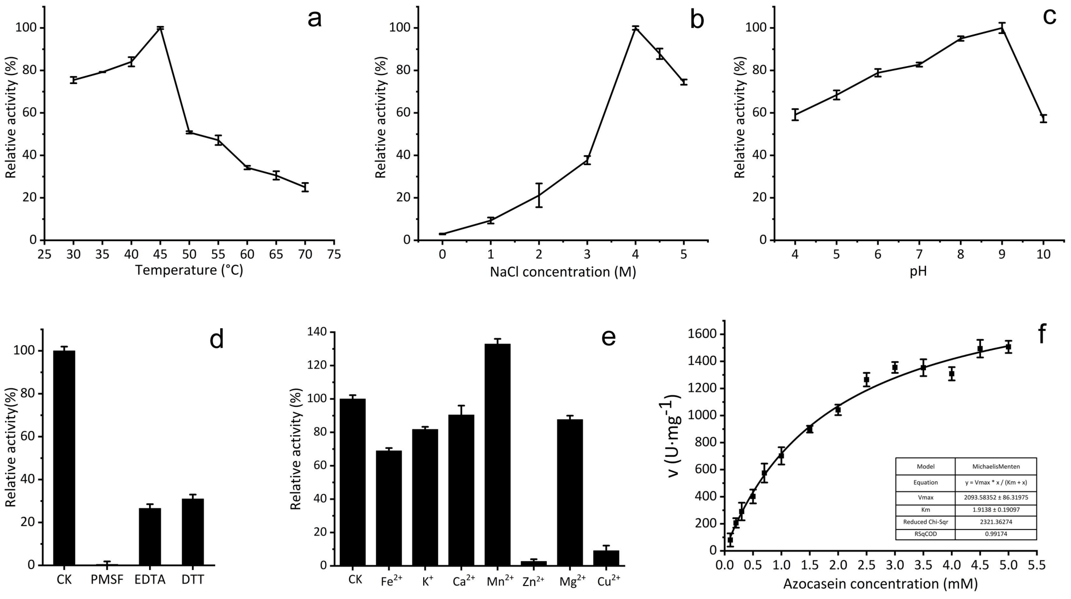

Fig. S5 Physicochemical properties and enzyme kinetics of halolysin HlyR4. The single-actor experiments of temperature (a), NaCl concentration (b) and pH value (c) for exerting optimal enzyme activity were tested. Three organic reagents, i.e., PMSF (phenylmethanesulfonyl fluoride), EDTA (ethylene diamine tetraacetic acid) and DTT (dithiothreitol) (d), and seven inorganic reagents, i.e., Fe^{2+}, K^{+}, C^{2+}, Mn^{2+}, Zn^{2+}, Mg^{2+} and Cu^{2+} (e), were selected to probe its impact on enzyme activity. Enzyme kinetics including V_{max}, K_{m} and K_{cat} of HlyR4 were determined in different azocasein concentrations (f).

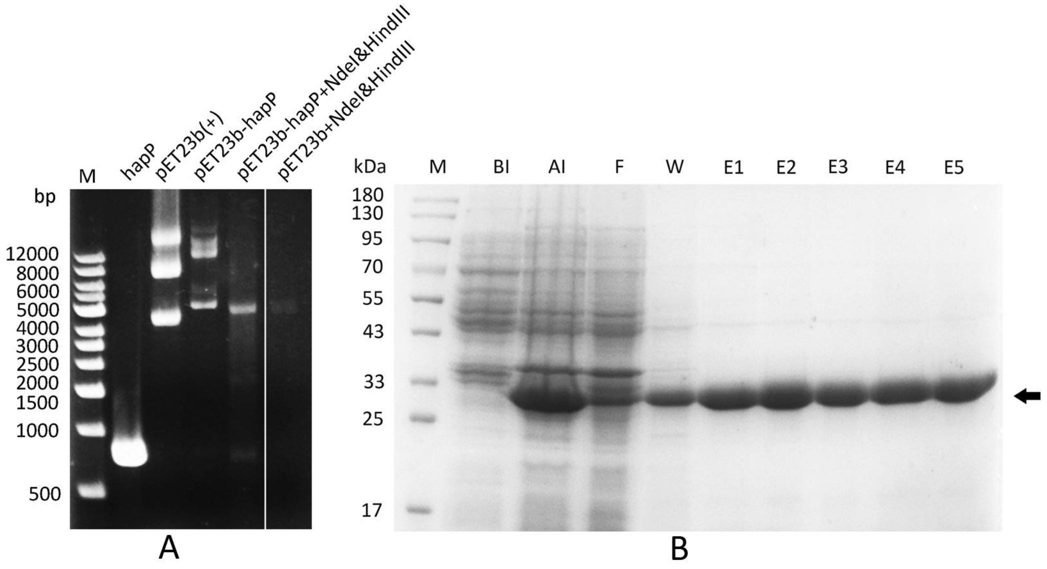

Fig. S6 Plasmid construction and gene expression of *hapP*. The *hapP* was inserted into the expression plasmid pET23b(+) resulting in the recombinant plasmid pET23b-hapP. The plasmid pET23b-hapP was verified through double (*Nde*I and *Hin*dIII) enzyme digestion (A) as well as DNA sequencing before protein expression. The plasmid pET23b-hapP was transformed into *E. coli* BL21 (DE3) for protein expression with IPTG induction (B). BI, Before IPTG induction; AI, after IPTG induction; F, flow through solution; W, washing through solution; E1-E5, elutes; arrow shows the target products, HmHapP. The DNA ladder and protein molecular standard are shown on the left.

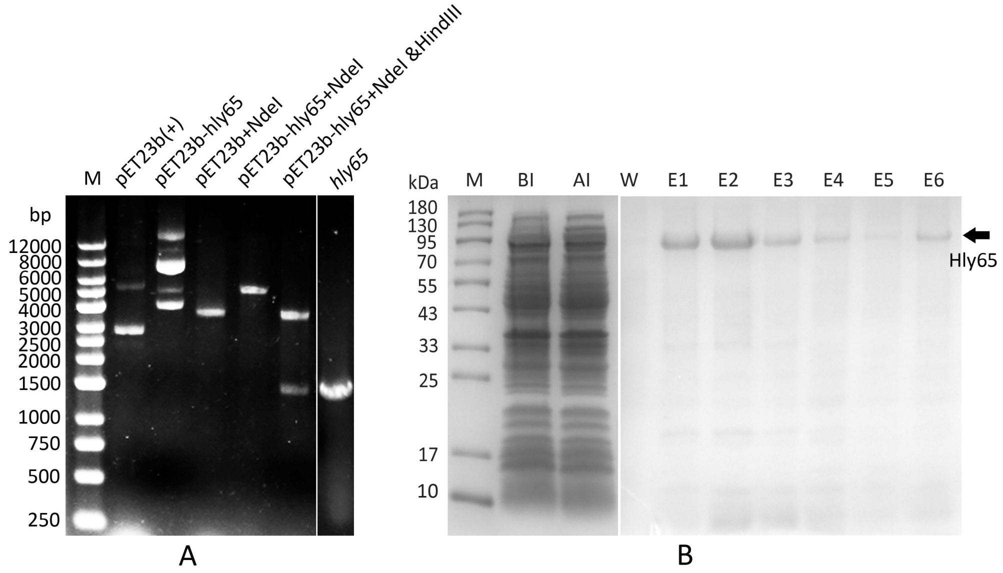

Fig. S7 Plasmid construction and gene expression of *hly65*. The *hly65* was inserted into the expression plasmid pET23b(+) resulting in the recombinant plasmid pET23b-hly65. The plasmid pET23b-hly65 was verified through single (*Nde*I) and double (*Nde*I and *Hin*dIII) enzyme digestions (A) as well DNA sequencing before protein expression. The plasmid pET23b-hly65 was transformed into *E. coli* BL21 (DE3) for protein expression with IPTG induction. The effect of gene expression and protein purification through Ni-NTA affinity chromatography was checked by SDS-PAGE (B). BI, Before IPTG induction; AI, after IPTG induction; W, washing through solution; E1-E6, elutes; arrow shows the target products, Hly65. The DNA ladder and protein molecular standard are shown on the left.

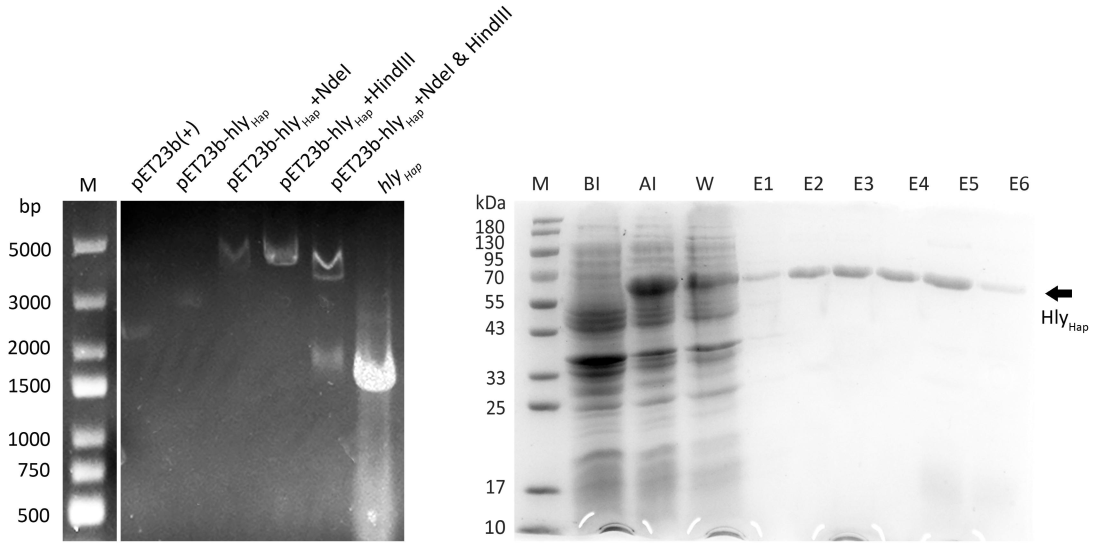

A B

Fig. S8 Plasmid construction and gene expression of *hly*$_{\mathbf{Hap}}$. The *hly*$_{\text{Hap}}$ was inserted into the expression plasmid pET23b(+) resulting in the recombinant plasmid pET23b-hly$_{\text{Hap}}$. The plasmid pET23b-hly65 was verified through single (*Nde*I or *Hin*dIII) and double (*Nde*I and *Hin*dIII) enzyme digestions (A) as well DNA sequencing before protein expression. The plasmid pET23b-hly$_{\text{Hap}}$ was transformed into *E. coli* BL21 (DE3) for protein expression with IPTG induction. The effect of gene expression and protein purification through Ni-NTA affinity chromatography was checked by SDS-PAGE (B). BI, Before IPTG induction; AI, after IPTG induction; W, washing through solution; E1-E6, elutes; arrow shows the target products, Hly$_{\text{Hap}}$. The DNA ladder and protein molecular standard are shown on the left.

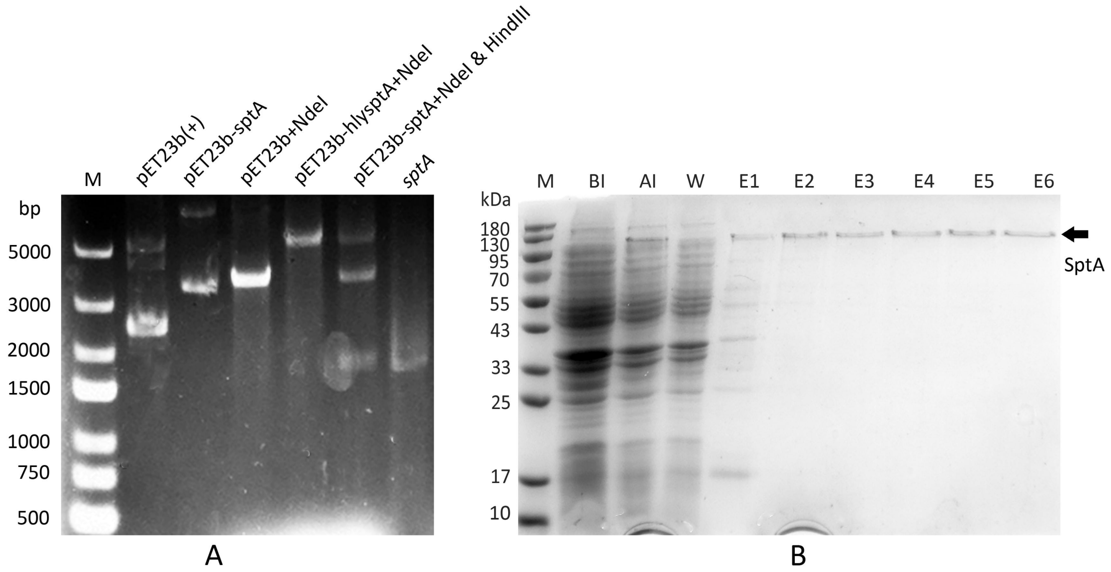

Fig. S9 Plasmid construction and gene expression of *sptA*. The *sptA* was inserted into the expression plasmid pET23b(+) resulting in the recombinant plasmid pET23b-sptA. The plasmid pET23b-sptA was verified through single (*Nde*I) and double (*Nde*I and *Hin*dIII) enzyme digestions (A) as well DNA sequencing before protein expression. The plasmid pET23b-sptA was transformed into *E. coli* BL21 (DE3) for protein expression with IPTG induction. The effect of gene expression and protein purification through Ni-NTA affinity chromatography was checked by SDS-PAGE (B). BI, Before IPTG induction; AI, after IPTG induction; W, washing through solution; E1-E6, elutes; arrow shows the target products, SptA. The DNA ladder and protein molecular standard are shown on the left.

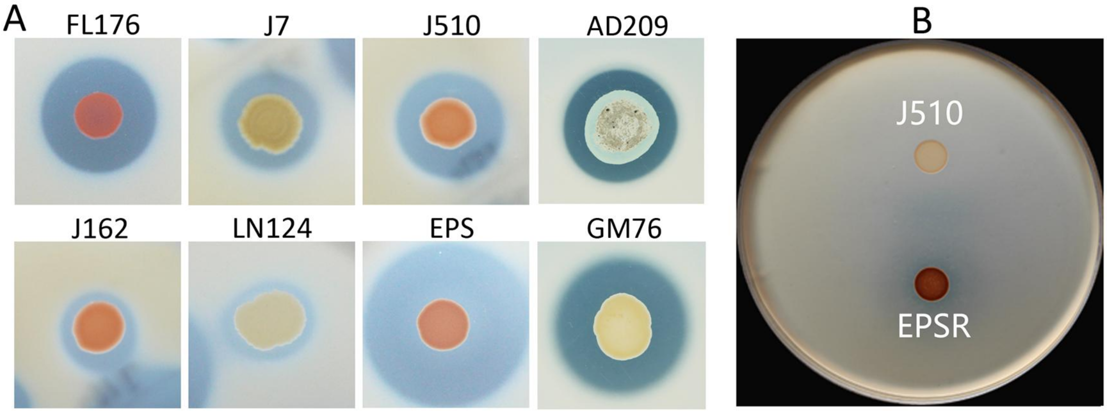

Fig. S10 Proteolytic activity of strains and synergistic antagonism between strains J510 and EPSR. (A) Proteolytic activity detection on JCM 168 skim-milk agar plate. FL176, *Haloarchaeobius* sp. FL176; J7, *Natrialba* sp. J7; J510, *Halococcus* sp. J510; AD209, *Saccharomonospora* sp. AD209; J162, *Halococcus* sp. J162; LN124, *Pseudomonas* sp. LN124; EPS, *Haloferax mediterranei* EPS. (B) Synergistic antagonism between strains J510 and EPSR on LN72 plate. EPSR, *Haloferax mediterranei* EPSR, a hlyR4 deficient EPS strain.

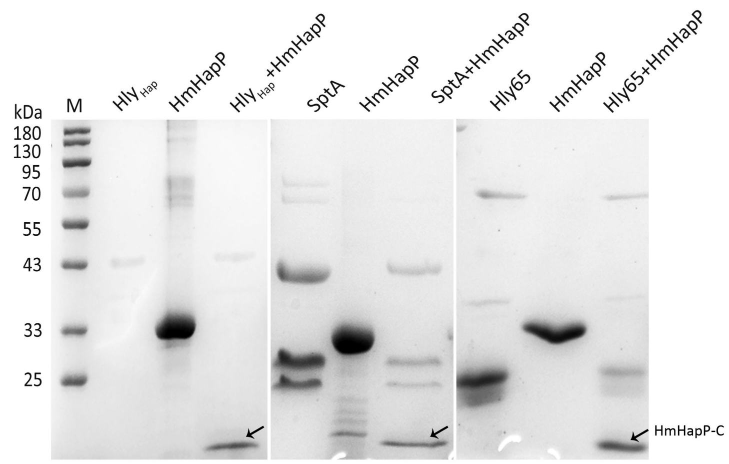

Fig. S11 Cleavage of HmHapP by halolysins Hly$_{Hap}$, SptA and Hly65. Proteins Hly$_{Hap}$, SptA and Hly65 were expressed in *E. coli* BL21 (DE3) and purified using Ni-NTA affinity chromatography. Cleavage reaction was conducted by mixing refolded HmHapP and halolysins. Trichloroacetic acid (TCA) precipitation was conducted prior to performing sodium dodecyl sulfate polyacrylamide gel electrophoresis (SDS-PAGE). HmHapP-C, the products of HmHapP cleaved by halolysins (↙).

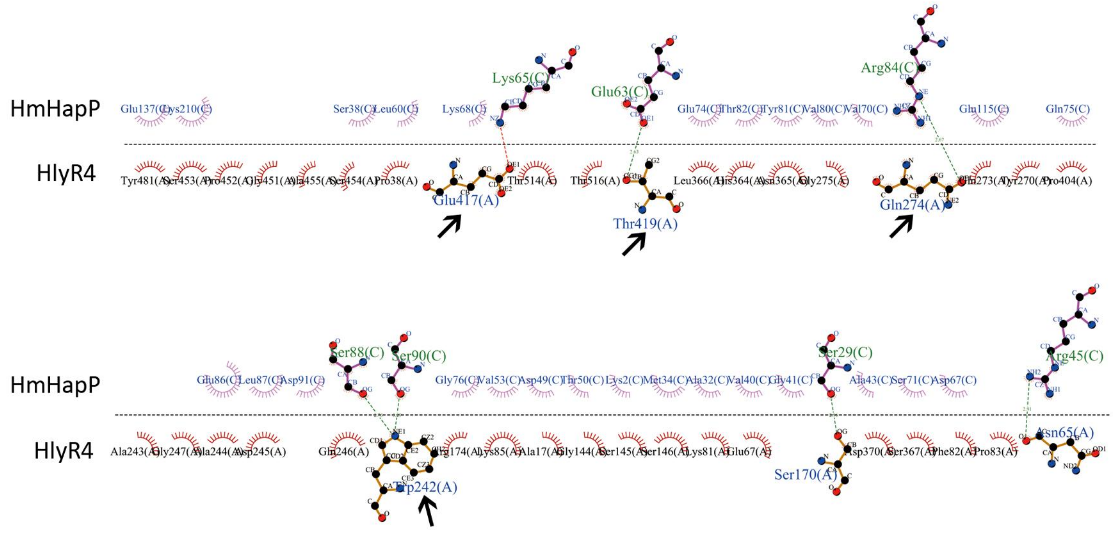

Fig. S12 Protein-Protein Docking with AlphaFold-Multimer. Four possible interaction sites, Trp^{242}, Gln^{274}, Glu^{417} and Thr^{419} (black arrows) were predicted in the contact interface at the side of HlyR4 using AlphaFold-Multimer. Amino acid residues Ser^{170} and Asn^{65} were not chosen for further analysis due to far away from the contact interface.

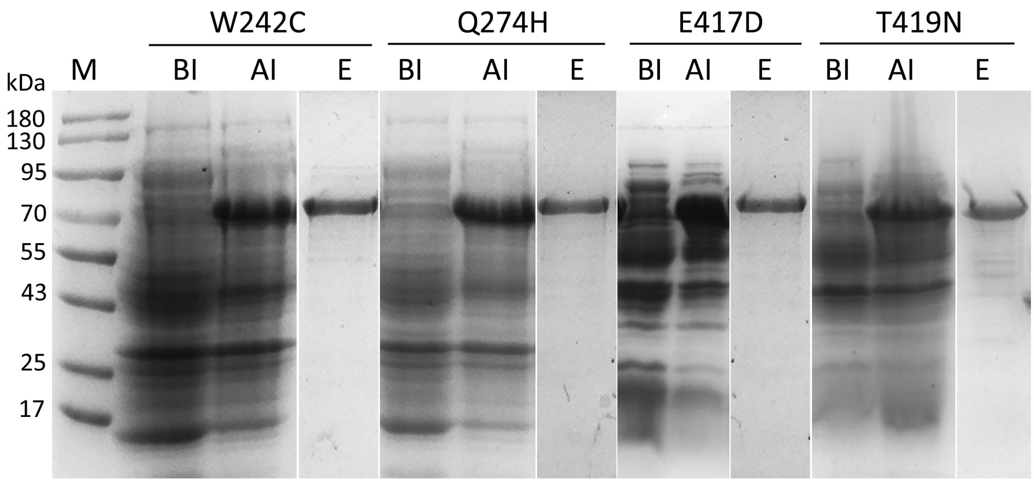

Fig. S13 Gene expression and protein purification of four HlyR4 point mutation mutants. Four HlyR4 point mutation mutants, W242C, Q274H, E417D and T419N, were purified using the Ni-NTA affinity chromatography. BI, Before IPTG induction; AI, after IPTG induction; E, elutes. The protein molecular standard is shown on the left.

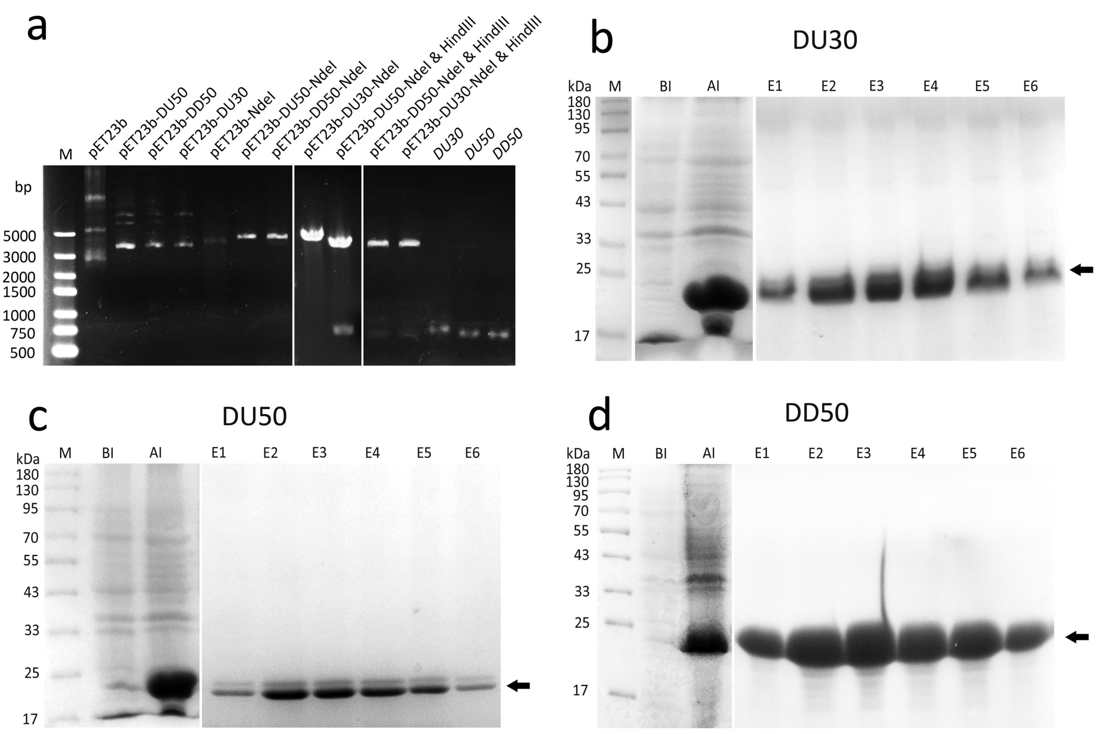

Fig. S14 Plasmid construction, protein expression and purification of the HmHapP truncated proteins. The *hapP* without its 5'-terminal 90 bp (*DU30*), without the 5'-terminal 150 bp (*DU50*) and without the 3'-terminal 150 bp (*DD50*) were inserted into the expression vector pET23b(+) at the *Nde*I and *Hind*III sites, resulting in the recombinant plasmids pET23b-DU30, pET23b-DU50 and pET23b-DD50, respectively (a). The PCR products of *DU30*, *DU50* and *DD50* were taken as the controls (a). And these recombinant plasmids were checked using single enzyme digestion and double enzymes digestion (a) as well DNA sequencing before protein expression. Protein expression and purification of DU30 (b), DU50 (c) and DD50 (d) were conducted using Ni-NTA affinity chromatography. BI, Before IPTG induction; AI, after IPTG induction; F, flow through solution; W, washing through solution; E1-E6, elutes; arrow shows the target products. The DNA ladder and protein molecular standard are shown on the left.

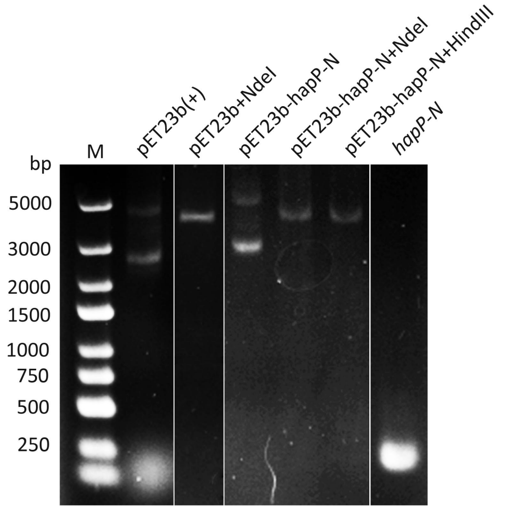

Fig. S15 Plasmid construction for expression of the *hapP-N*. The DNA sequence (135 bp) encodes the N-terminus (45 aa) of the HapP was entitled as *hapP-N*. The *hapP-N* was inserted into the plasmid pET23b(+) at the restriction sites of *Nde*I and *Hin*dIII via gene synthesis approach resulting in pET23b-hapP-N under codon optimization. Plasmid pET23b-hapP-N was checked through enzyme digestion using *Nde*I or *Hin*dIII as well DNA sequencing before protein expression. The DNA ladder is shown on the left.

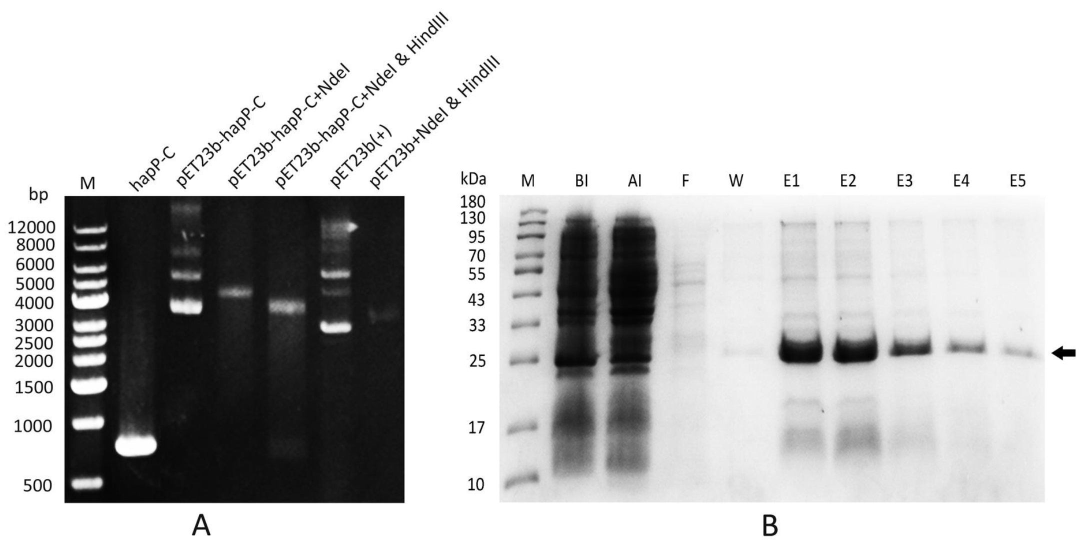

Fig. S16 Plasmid construction and gene expression of the *hapP-C*. The DNA sequence (570 bp) encodes the C-terminus (190 aa) of the HapP was named as *hapP-C*. The *hapP-C* was inserted into the plasmid pET23b(+) at the restriction sites of *Nde*I and *Hin*dIII resulting in pET23b-hapP-C. Plasmid pET23b-hapP-C was checked via single (*Nde*I) and double (*Nde*I and *Eco*RI) enzyme digestions (A) as well DNA sequencing before gene expression. The plasmid pET23b-hapP-C was transformed into *E. coli* BL21 (DE3) for protein expression with IPTG induction (B). BI, Before IPTG induction; AI, after IPTG induction; F, flow through solution; W, washing through solution; E1-E5, elutes. The black filled arrow shows the target product, HmHapP-C. The DNA ladder and protein molecular standard are shown on the left of the gel.

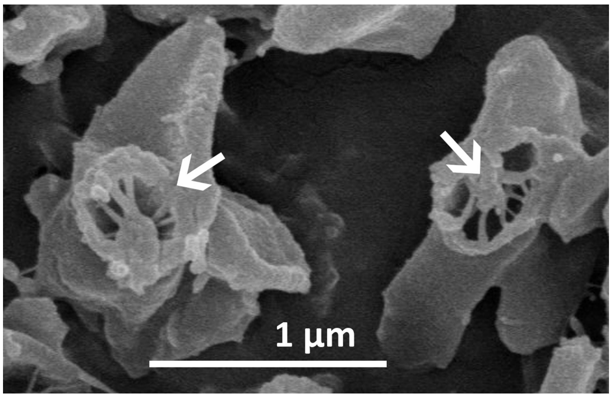

Fig. S17 Enlarged view of the perforated cells. The white arrow shows the wheel-like hole on the cell wall. Bar, 1 μm.

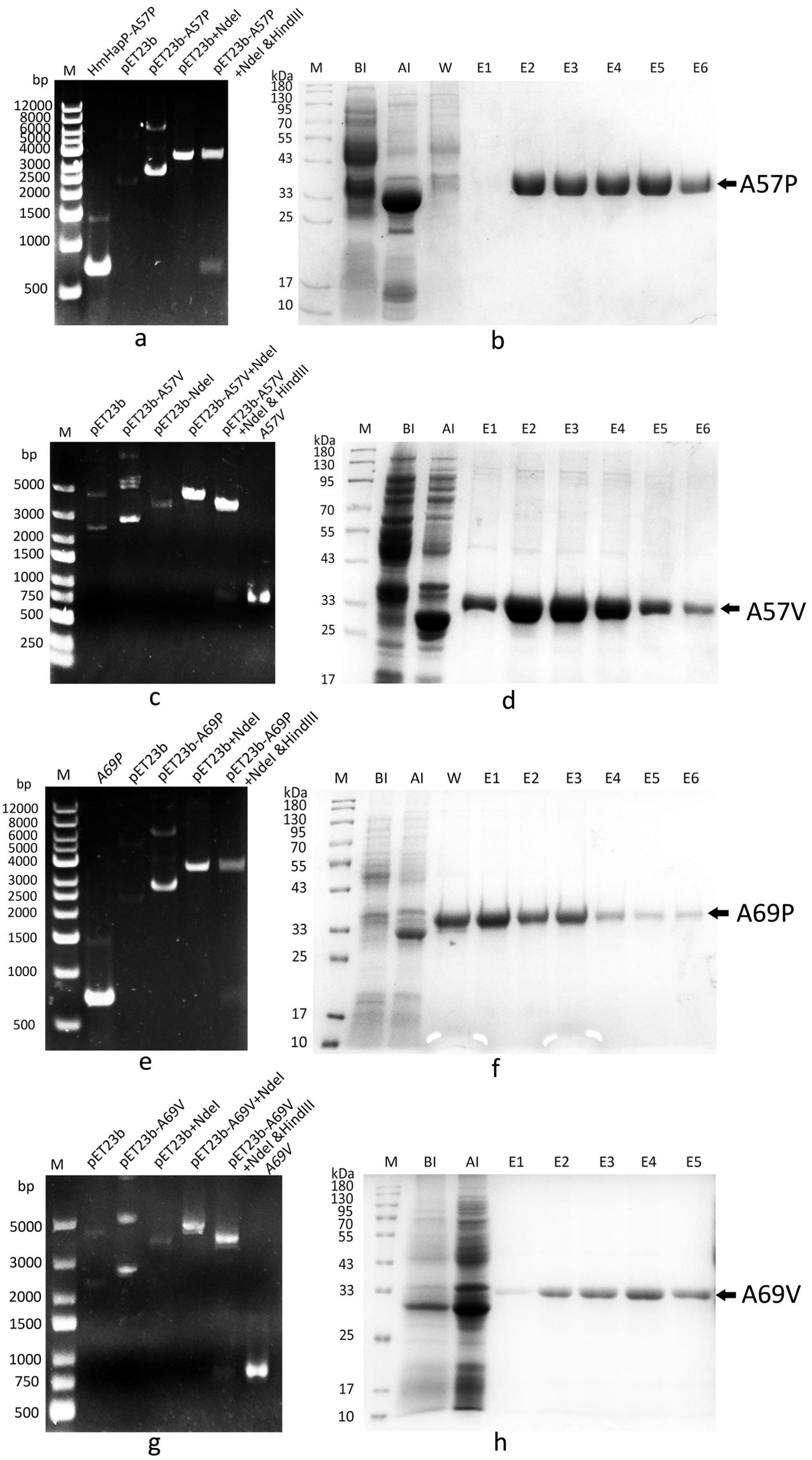

Fig. S18 Plasmid construction, verification, gene expression and protein purification of A57P, A57V, A69P and A69V. Plasmid construction and verification are shown on the left column. The type II restriction enzymes *Nde*I or/and *Hin*dIII were used to confirm the recombinant plasmids (a, c, e and g). Gene expression and protein

purification are shown on the right column. Proteins were purified using Ni^{2+} affinity chromatography. Target protein is shown in arrow (b, d, f and h). BI, Before IPTG induction; AI, after IPTG induction; F, flow through solution; W, washing through solution; E1-E5 or E1-E6, elutes. DNA ladder and protein molecular standard are shown on the left of each gel.

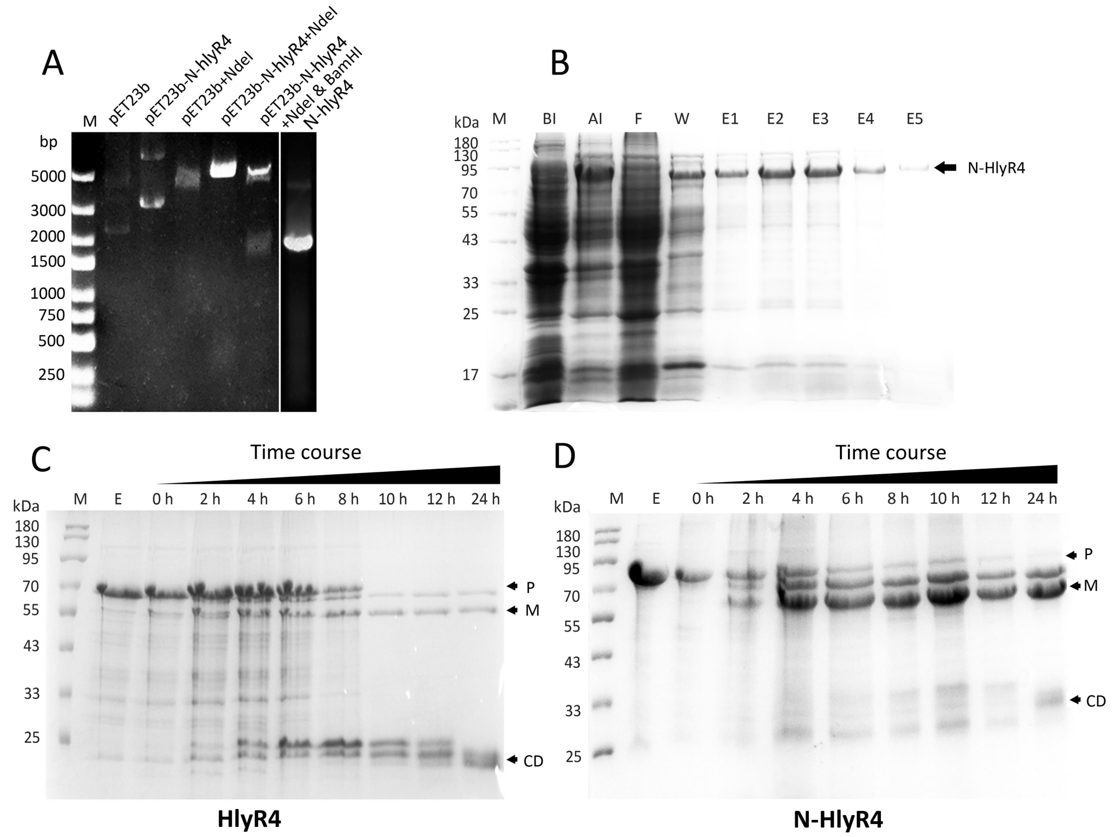

Fig. S19 Plasmid construction, gene expression of the *N-hlyR4* and refolding progress of N-HlyR4. (A) The DNA sequence (135 bp) encodes the HmHapP-N, *hapP-N,* was fused to the 5′-terminus of the *hlyR4* using overlapping PCR amplification resulting in the DNA fragment *N-hlyR4*. The *N-hlyR4* was inserted into the expression plasmid pET23b(+) at the restriction sites of *Nde*I and *Bam*HI, resulting in the recombinant plasmid pET23b-N-hlyR4. Single and double enzyme(s) digestion were applied to verify the correctness of the recombinant plasmid pET23b-N-hlyR4 before gene expression. The PCR product of *N-hlyR4* was taken as the control. (B) The plasmid pET23b-N-hlyR4 was transformed into *E. coli* BL21 (DE3) for gene expression with IPTG induction. The filled arrow shows the target protein, N-HlyR4. BI, Before IPTG induction; AI, after IPTG induction; F, flow through solution; W, washing through solution; E1-E5, elutes. (C) Time course of the refolding the halolysin HlyR4. Trichloroacetic acid (TCA) precipitation was conducted prior to performing sodium dodecyl sulfate polyacrylamide gel electrophoresis (SDS-PAGE). (D) Time course of the refoldingof the protein N-HlyR4. TCA precipitation was also performed prior to the SDS-PAGE. The time course for refolding is shown. P,

precursor; M, mature forms; CD, core domain of the mature protein. The DNA ladder or protein molecular standard are shown on the left of the gel.

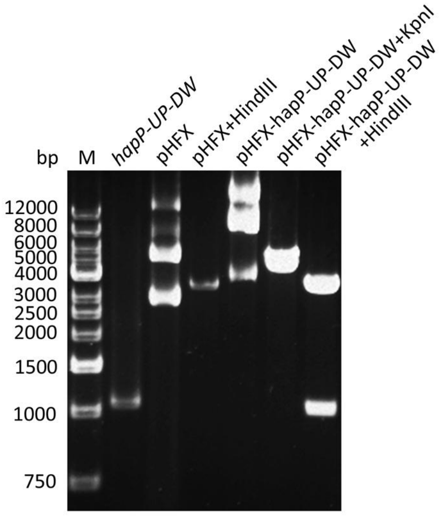

Fig. S20 Construction and verification of the *hapP* gene-knock-out plasmid. The DNA sequence up-stream (543 bp) and down-stream (537 bp) of the *hapP* was linked together via overlapping PCR plus a round of traditional PCR amplification resulting in the DNA fragment hapP-UP-DW. The hapP-UP-DW was inserted into the *E. coli*-haloarchaea shuttle vector pHFX at the restriction site of *Hin*dIII, resulting in the recombinant plasmid pHFX-hapP-UP-DW. Plasmid pHFX-hapP-UP-DW was verified using enzyme digestions by *Kpn*I or *Hin*dIII as well DNA sequencing before transformation.

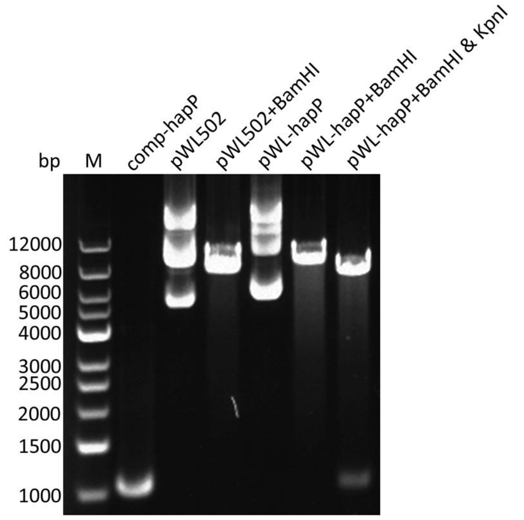

Fig. S21 construction and verification of the *hapP* gene complementary plasmid. The *hapP* (*HFX_0892*) as well as 150 bp DNA fragment up-stream was inserted into the *E. coli*-haloarchaea shuttle vector pWL502 at the restriction sites of *Bam*HI and *Kpn*I, resulting in the recombinant plasmid pWL-hapP. Plasmid pWL-hapP was verified through enzyme digestion with single enzyme digestion (*Bam*HI), or double enzyme digestion (*Bam*HI & *Kpn*I) as well DNA sequencing before transformation.

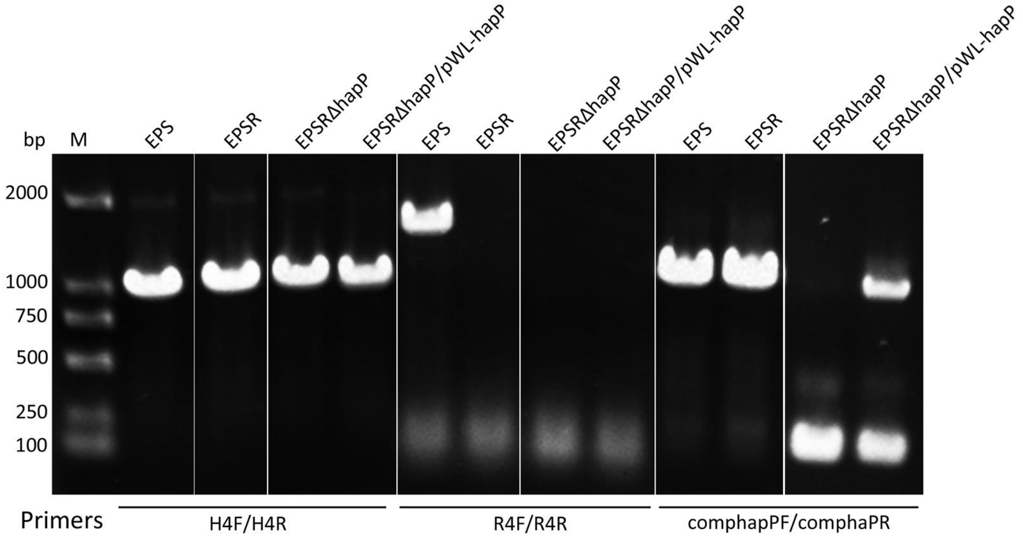

Fig. S22 Verification of the *hapP* gene knock-out and complementary strains. The primer pairs H4F/H4R, R4F/R4R and comphapPF/comphaPR were used to amplify the *halH4* (HFX_5264), *hlyR4* (HFX_2419) and *hapP* (HFX_0892), respectively. Strains EPS, EPSR, EPSRΔhapP and EPSRΔhapP/pWL-hap were derived from *Haloferax mediterranei* ATCC 33500. Primer names are shown at the bottom. Primer sequence is shown in Table 2. PCR products were verified through DNA sequencing.

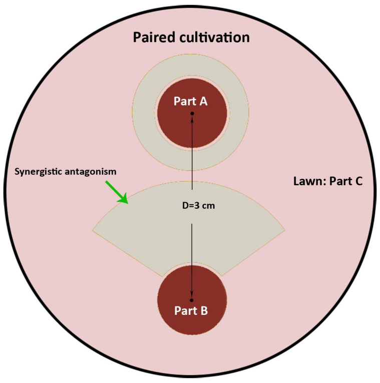

Fig. S23 The schematic diagram of paired cultivation. Part A, the extracellular protease production strain. Part B, the possible precursor proteins production strain, for example strain EPSR. Part C, strain used as lawn which is sensitive to the effective molecules produced by cleaving of precursor protein by extracellular protease. The synergistic antagonism phenomenon (fan-shaped sector) is shown in green arrow (↙). The center of the colony is shown in filled black circle (•). The distance (D) between part A and part B is 3 cm. The cultivation condition depends on the strains used for screening synergistic antagonism.

Table S1 Codon optimization[a]

Gene name	Original DNA	Optimized DNA
hapP<br><br>(*HFX_0892*)	AGACAACCAACGGACACCGATCATGTGCTTAAA<br>GAAGCAGAGGAACTGTTCGCGGAATGTAAGGT<br>CGACAAGGCAGTCAGTGCGATTGAGCAGGGAG<br>GAGGGACTGTCTATACTACCCGAAGCGAGCTCA<br>GCAAATCCGACTATAATCAAAGCGAGGCTAATG<br>AAAATGAAGTTGGAACCAGGGATGCTTCATACC<br>CAGAAAGCGACTCTGAATTCCGACTAGATTATTG<br>GACTACATCATCTCAGATAGGGGCAACCCTCAGT<br>ATGCACTTTGGCGAAGTAGTCTCGACAGCATAC<br>GGGGCAACCAAAGCTGAGGACGCAATGGGAAT<br>TACATTCAATGGTGATGCGTATAACCTAGACAAA<br>GCACCCGAACTCAGTGTGAGTCCACATGCAAAA<br>GAAGACTATGACTGGTCTATCAGGGCCTCAGATT<br>ACAACCCAGGCAAAGCGGGTGTGGCCGCAAGA<br>GTCGACACGTCTTGGGAGAAACGAGAGTACCC<br>AAAAGATGTAACCGTGGTGATGGTTATATACCTC<br>GAAAGTACAGCCAGTTCAAAACCACCTGTATTC<br>GGAGAATACAAGCATAATAACGCATTGACGGAC<br>GGTGGTCTCAAATCAATTAGCCTTGTCCCCCCCA<br>ATGGGGGAATCGGTGTTGAACTCGCGACATCTG<br>CATCAACTGTCTGGACCCAGTCTGGTTTTGCTGG<br>GAAAGACGCT	CGTCAGCCTACCGATACCGATCATGTGCTGAAAGAAG<br>CCGAAGAACTGTTTGCAGAATGCAAAGTGGATAAAG<br>CAGTTAGCGCCATTGAACAGGGTGGTGGCACCGTGT<br>ATACCACCCGTAGCGAACTGAGCAAAAGTGATTATAA<br>TCAGAGTGAAGCCAATGAAAATGAAGTGGGCACCCG<br>CGATGCAAGCTATCCGGAAAGCGATAGTGAATTTCGT<br>CTGGATTATTGGACCACCAGCAGTCAGATTGGCGCA<br>ACCCTGAGCATGCATTTTGGCGAAGTTGTGAGCACC<br>GCCTATGGCGCAACCAAAGCCGAAGATGCAATGGGT<br>ATTACCTTTAATGGCGATGCCTATAATCTGGATAAAGC<br>CCCGGAACTGAGTGTGAGTCCGCATGCAAAAGAAGA<br>TTATGATTGGAGTATTCGTGCAAGCGATTATAATCCGG<br>GCAAAGCAGGCGTTGCAGCACGTGTTGATACCAGTT<br>GGGAAAAACGCGAATATCCGAAAGATGTGACCGTGG<br>TTATGGTTATCTATCTGGAAAGTACCGCCAGCAGCAA<br>ACCGCCGGTGTTTGGTGAATATAAACATAATAATGCG<br>CTGACCGATGGTGGTCTGAAAAGTATTAGTCTGGTTC<br>CGCCGAATGGTGGTATTGGTGTGGAACTGGCCACCA<br>GCGCCAGCACCGTGTGGACCCAGAGTGGCTTTGCCG<br>GTAAAGATGCA
hly65<br><br>(PP992913)	ACCCCCGGACGCAGTCCGGGACCGAAGGACGA<br>CGAGGTACTCGTCGGCGTCTCGGCGAGCGCCG<br>ACCTCCGGAAGACCGTCGAACAGCACGTCCCCG<br>GCAACGCCGAGGTCGTCCACCAGAACGACGAC<br>CTCCGGTACGTCGCGGTGAAGTTCAAGGGCAG<br>CGACACTGCACGCGAGAACTTCAAGGACGCGA<br>TCGCGAAGAAAGAGCACGTCAAGTACGCAGAG<br>GACAACGCGACGTTCCACGCGCTCGCGACGCC<br>GAACGACCCCAAGTTCGGCCAGCAGTACGCACC<br>CCAGCAGGTCAACTCCGACCAGGCCTGGGACA<br>CCACGTTCGGCGACTCGAACGTCACCATCGCGG<br>TCGTCGACACCGGCGCACAGTACTCTCACCCCG<br>ACCTCTCCGGGAACTACGAGTCCGACCCGGGCA<br>AGGACTTCGTCGACAACGACGCCGACCCGGCG<br>CCGGACGTCCCGAGCGACGAGTATCACGGCAC<br>ACACGTCTCCGGGTGCGCCGCCGCGGTCGTCGA<br>CAACGGTACCGGCGTCGCCGGCCAGGGTAACTC<br>CTCGCTCATCAACGGTCGTGCGCTCGACGAGTC<br>CGGCGGCGGGAGCCTCTCGGACATCGCCGACG<br>CCGTCAAGTGGGCGGCCGACCAGGGCGCGGAC<br>GTCATCAACATGAGCCTCGGCGGCGGCGGTTAC<br>ACGGACACGATGAAGAACGCCGTCAGCTACGC<br>GACGAACAACGGCTCGCTCGTCTTCGCTGCCGC<br>CGGGAACGACGGCAGCCAGGGCGTCTCCTATCC<br>CGCGGCGTACAGCGAGTGCGTCGCCGTCTCCGC<br>GGTCGACGACAGCGAGAACCTCGCGAGCTTCA<br>GCCAGTACGGCTCCAGCGTCGAACTGTGCGCGC<br>CCGGCGTCGACGTCCTCTCGACGACCACCGAGA<br>CCCGCGGCAGCTACGAGACGCTCTCGGGGACG<br>TCGATGGCGACCCCCGTCACGTCCGGCGTCGCG<br>GGTCTCACGCTCGCGAAGTGGAGCGGCCTCAC<br>GAACAACGAACTCCGCAGCCACCTGAAGAACA<br>CCGCCGCGGACATCGGGCTCTCCAGCGACGAG<br>CAGGGGAGCGGGCAGGTCGACGCGTACGCCGC<br>AGTCACCACGGACCCGTCCGGCGGCGACGACG<br>GCGGTGACGGCGGTGGCGGTGGCGGCGACTCC<br>ACGTCCAGTTCGCTCTCGGGGTCGCTCTCGGGC<br>TACTACGACTACGACGACTACAGCTACGGCTGG<br>AACTACTCCTCGCCGAGCCAGGTCGTCGTCGAG<br>CTCGACGGCCCGAGCGACGCCGACTTCGACCTC<br>TACGTGAACACGGGGACGACGGCGGCCGCGAC<br>GCCGTCGGACTACGACTACGCGTCGTACACCGC<br>CGACAGTCAGGAGACAATCACCATCGACAACCC<br>CGACGACTCGACGGACTTGCAGGTCGACGTGG<br>ACTCCTACAGCGGCAGCGGGAGCTACACGCTGA<br>CCATCACGGAGTACCAG	ACCCCTGGTCGCAGTCCGGGTCCGAAAGATGATGAA<br>GTGCTGGTGGGTGTTAGCGCAAGCGCAGATCTGCGT<br>AAAACCGTGGAACAGCATGTGCCGGGCAATGCCGAA<br>GTGGTTCATCAGAATGATGATCTGCGCTATGTTGCAG<br>TGAAATTCAAAGGTAGTGATACCGCACGCGAAAATTT<br>TAAAGATGCAATTGCAAAAAAGGAGCATGTTAAATAT<br>GCAGAAGATAATGCCACCTTTCATGCCCTGGCAACCC<br>CGAATGATCCGAAATTTGGTCAGCAGTATGCCCCGCA<br>GCAGGTGAATAGTGATCAGGCCTGGGATACCACCTTT<br>GGCGATAGTAATGTTACCATTGCAGTTGTTGATACCG<br>GCGCACAGTATAGCCATCCGGATCTGAGCGGCAATTA<br>TGAAAGTGATCCGGGCAAAGATTTTGTTGATAATGAT<br>GCAGATCCGGCACCGGATGTTCCGAGCGATGAATATC<br>ATGGCACCCATGTGAGCGGTTGTGCCGCAGCAGTTG<br>TTGACAATGGTACCGGTGTTGCAGGTCAGGGCAATA<br>GCAGCCTGATTAATGGCCGCGCACTGGATGAAAGCG<br>GTGGTGGTAGCCTGAGCGATATTGCAGATGCAGTTAA<br>ATGGGCCGCAGATCAGGGTGCAGATGTGATTAATATG<br>AGCCTGGGTGGTGGTGGCTATACCGATACCATGAAAA<br>ATGCCGTGAGTTATGCAACCAATAATGGTAGTCTGGT<br>TTTTGCCGCAGCAGGTAATGATGGTAGCCAGGGTGT<br>GAGTTATCCGGCCGCATATAGTGAATGTGTTGCAGTG<br>AGCGCCGTGGATGATAGTGAAAATCTGGCAAGTTTTA<br>GTCAGTATGGTAGTAGCGTGGAACTGTGTGCCCCGG<br>GCGTGGATGTGCTGAGCACCACCACCGAAACCCGCG<br>GTAGCTATGAAACCCTGAGCGGCACCAGTATGGCAA<br>CCCCGGTGACCAGCGGTGTTGCAGGCCTGACCCTGG<br>CAAAATGGAGCGGCCTGACCAATAATGAACTGCGCA<br>GCCATCTGAAAAATACCGCCGCCGATATTGGTCTGAG<br>CAGTGATGAACAGGGTAGCGGCCAGGTTGATGCCTA<br>TGCCGCAGTGACCACCGATCCGAGTGGTGGTGACGA<br>TGGCGGCGATGGTGGTGGCGGTGGTGGTGACAGTA<br>CCAGCAGCAGCCTGAGCGGCAGTCTGAGCGGTTATT<br>ATGATTATGATGATTATAGCTACGGCTGGAATTATAGTA<br>GCCCGAGCCAGGTTGTTGTTGAACTGGATGGCCCGA<br>GTGATGCAGATTTTGATCTGTATGTTAATACCGGCACC<br>ACCGCAGCAGCCACCCCGAGTGATTATGATTATGCAA<br>GTTATACCGCCGATAGCCAGGAAACCATTACCATTGAT<br>AATCCGGATGATAGTACCGATCTGCAGGTTGATGTTG<br>ATAGTTATAGCGGCAGCGGCAGCTATACCCTGACCATT<br>ACCGAATATCAG
sptA<br><br>(AY800382.<br><br>1)	ACGCCGGGACGGGAGCCGGGGCCGAAGAAAG<br>ATGAGCTGATCGTCGGGGTCGACCCCGACGTCT<br>CGAACATCGAAGCGGCCGTGGAGCCGAAGATT<br>CCGAGCAACGCGAACATTGTCCACACGAACGA<br>GACGCTGGGCTATGCCGCAGTGGAGATCGCCGA<br>TCAGGCTTCCATTCAGGCCAAGGAATCCGTCAA<br>GCGGAGCGTCCTCGATGCCGACGAGGTGACCTA<br>CTCCGAAGACAACGTGACCTACGAGGCCATCGA<br>GGCGGAACCACAGGAATTGGAAAGCGATGGCG<br>AGACGGCGTCGCCGCTCTATACCCCGAACGACC<br>CGGACTTCGGGAGTCAGTACGCGCCACAGCAG	ACCCCTGGTCGCGAACCGGGTCCGAAAAAAGATGAA<br>CTGATTGTTGGTGTTGATCCGGATGTTAGTAATATTGA<br>AGCCGCCGTGGAACCGAAAATTCCGAGTAATGCCAA<br>TATTGTTCATACCAATGAAACCCTGGGTTATGCCGCAG<br>TTGAAATTGCAGATCAGGCCAGTATTCAGGCAAAAG<br>AAAGCGTTAAACGTAGCGTGCTGGATGCCGATGAAG<br>TTACCTATAGCGAAGATAATGTTACCTATGAAGCAATT<br>GAAGCAGAACCGCAGGAACTGGAAAGCGATGGTGA<br>AACCGCAAGTCCGCTGTATACCCCGAATGATCCGGAT<br>TTTGGCAGTCAGTATGCACCGCAGCAGGTGAATGCC<br>CCGGAAGCCTGGAATACCACCCTGGGCGATCCGGAA

	GTGAACGCGCCGGAAGCCTGGAACACGACCCT<br>CGGCGATCCGGAGGTTACGATCTCGATCGTCGA<br>CCAAGGGGTCCAGTACGACCACCCGGATCTCGC<br>GGAGAACATGGACAACAGCGTCTCGAACGGTG<br>GGTCCGACTTCGTCGATGATAACGGCGATCCGT<br>ACCCGGCAGACGCGAGCGAGAACCACGGGAC<br>GCACGTGGCTGGGATCGCAGCCGGTGGCACCG<br>ACAACGGGACGGGCCATGCCGGTATTTCGAACT<br>GTTCGCTGCTCTCGGCCCGCGCGCTCGGTGGCG<br>GGGGCAGCGGTTCGCTCTCCGACATCGCCGATG<br>CGGTCCAGTGGTCGGCCGACCAGGGTGCCGAC<br>ATCATCAACATGTCCCTCGGTGGCGGCGGTGCT<br>ACGCAACTGATGCGGGAGGCCTGTGAGTACGC<br>CGCCTCGCAGGGGACATTGGTCGTCGCAGCGG<br>CCGGTAACGACTACGGCAGCAGCGTCTCGTACC<br>CGGCCGCCTACGACACCGTCCTCGCCGTCTCCTC<br>GCTGGACCAGGGTGAAACGCTGTCCGACTTCTC<br>GAACGTCGGCCCGGAAATCGAACTGGCCGCAC<br>CCGGTGGCAACGTCCTCTCGAGCGTTCCCTGGG<br>GCGACTACGAGACCCTCTCCGGTACCTCGATGG<br>CGTCGCCGGTCGTCGCCGGCGTCGCCGGACTCA<br>CGCTCTCGGCGTGGCCGAACCTCTCGAACGATC<br>AGCTGCGAGACCATCTCAAGCAAACCGCCGTCG<br>ATGTCGGCCTTTCGGCGAACGAACAGGGCAGC<br>GGCCGCGTTGACGCCGGCAATGCCGTCACCACC<br>GAACCGGGTACCTCTCCGGACCCCGACCCGGA<br>GCCCGGTAAGTGCGGCGACGAGGTCAACACTG<br>CAAGCGAAGAGGGCGAACTCAGTGGTGGCTGG<br>GGCGGCAACCCGAACGATACCTACACCTACCAG<br>CTTCAGACGTCCGACCCCTGCAGTGCCACCGTC<br>TCGCTCGAGGGACCGGCCGACGCTGACTTCGA<br>CCTCTACATGACGCTCGACGGACGGACGCCGTC<br>GATGTACGACTACGACGAGCGCTCGACGGGCCA<br>GGGCGCAAGCGAAACGATCGAACTCGACCTCA<br>CAGGCGACGAAGAGTTAGGTGTCCTCGTCACCC<br>GGTACAGCGGAAGCGGCTCCTACTCCATGACTA<br>TCGACGAACGCGGTCGA	GTTACCATTAGCATTGTTGATCAGGGTGTGCAGTATG<br>ATCATCCGGATCTGGCAGAAAATATGGATAATAGTGT<br>GAGTAATGGTGGCAGCGATTTTGTGGATGATAATGGC<br>GATCCGTATCCGGCAGATGCAAGTGAAAATCATGGCA<br>CCCATGTTGCAGGCATTGCCGCAGGTGGTACAGATAA<br>TGGCACCGGCCATGCCGGCATTAGTAATTGTAGTCTG<br>CTGAGTGCCCGCGCACTGGGCGGCGGTGGTTCAGGT<br>AGCCTGAGCGATATTGCAGATGCCGTGCAGTGGAGC<br>GCCGATCAGGGCGCTGATATTATTAATATGAGCCTGG<br>GCGGCGGCGGTGCAACCCAGTTAATGCGCGAAGCAT<br>GCGAATATGCCGCCAGTCAGGGTACACTGGTTGTGG<br>CCGCAGCCGGTAATGATTATGGTAGTAGTGTTAGCTAT<br>CCGGCCGCATACGATACCGTTCTGGCCGTTAGCAGTC<br>TGGATCAGGGTGAAACCCTGAGTGATTTTAGCAATGT<br>GGGCCCGGAAATTGAACTGGCAGCACCGGGTGGCA<br>ATGTTCTGAGTAGTGTTCCGTGGGGCGATTATGAAAC<br>CCTGAGCGGCACCAGTATGGCCAGTCCGGTTGTTGC<br>AGGCGTGGCAGGCCTGACCCTGAGTGCATGGCCGAA<br>TCTGAGCAATGATCAGCTGCGTGATCATCTGAAACAG<br>ACCGCAGTGGATGTGGGCCTGAGCGCCAATGAACAG<br>GGTAGTGGCCGCGTGGATGCCGGCAATGCCGTTACC<br>ACCGAACCGGGCACCAGCCCGGACCCGGACCCGGA<br>GCCTGGTAAATGTGGTGACGAAGTTAATACCGCCAGC<br>GAAGAAGGTGAACTGAGTGGTGGCTGGGGTGGCAA<br>TCCGAATGATACCTATACCTATCAGCTGCAGACCAGTG<br>ATCCGTGTAGCGCCACCGTGAGCCTGGAAGGCCCGG<br>CAGATGCCGATTTTGATCTGTATATGACCCTGGATGGT<br>CGTACCCCGAGCATGTATGATTATGATGAACGTAGCA<br>CCGGCCAGGGTGCAAGTGAAACCATTGAACTGGATC<br>TGACCGGTGACGAAGAACTGGGTGTGCTGGTGACCC<br>GTTATAGTGGCAGCGGCAGCTATAGCATGACCATTGA<br>TGAACGTGGTCGT

[a]The original DNA sequence did not contain its signal peptide encoding sequence, and the different nucleotides after codon optimization were marked in red. The codon optimization of the DNA fragments *hapP-N* and *hapP-C* encoding the HmHapP-N (the N-terminal 45 aa of HmHapP) and HmHapP-C (the C-terminal 190 aa of HmHapP), respectively, were not shown, because they were identical to the *hapP*.

Table S2 The predicted functions of these 30 genes with the greatest change of transcriptional level in strains EPS, EPSR and EPSRΔ*hapP*.

Gene ID	Count			Description
	EPS	EPSR	EPSRΔ*hapP*	
HFX_2459	12094	11698	37398	BGTF surface domain-containing protein
HFX_2183	165	202	7348	copper-containing nitrite reductase
HFX_1927	4238	1400	6981	universal stress protein
HFX_2346	1372	293	2464	universal stress protein
HFX_5218	9832	291	7553	AbrB/MazE/SpoVT family DNA-binding domain-containing protein
HFX_1523	19323	2921	22172	SDR family oxidoreductase
HFX_1243	2731	678	3700	polymer-forming cytoskeletal protein
HFX_2178	2910	694	3708	glutamate dehydrogenase GdhB
HFX_0422	2741	258	2025	thermosome subunit beta
HFX_2388	1348	340	1654	CDC48 family AAA ATPase
HFX_5219	3734	166	1705	PHA granule structural protein PhaP
HFX_2049	1659	385	1963	aconitate hydratase
HFX_6329	2092	268	1709	urocanate hydratase
HFX_0232	2570	1559	3341	hypothetical protein
HFX_6257	851	778	829	type IV pilin N-terminal domain-containing protein
HFX_1352	4370	2527	5010	DJ-1/PfpI family protein
HFX_0082	12517	7752	12074	hypothetical protein
HFX_1696	7894	5848	6513	gas vesicle structural protein GvpA
HFX_2876	5649	2563	6660	DUF4013 domain-containing protein
HFX_5226	17570	8389	4277	hypothetical protein
HFX_0560	1902	746	2100	cell division protein FtsZ
HFX_6014	2033	378	622	superoxide dismutase
HFX_2009	1930	605	1871	cation: proton antiporter
HFX_2220	17210	4753	11040	PGF-pre-PGF domain-containing protein
HFX_0742	1472	486	1738	ATP-dependent protease LonB
HFX_1340	1828	336	1523	halocyanin domain-containing protein
HFX_0511	1386	380	1684	aconitate hydratase AcnA
HFX_6076	30818	3540	9878	S8 family peptidase
HFX_0738	7592	963	3423	thermosome subunit alpha
HFX_6412	3916	6	9	hypothetical protein
